# Supplementary material for: Therapeutic itineraries of snakebite victims and antivenom access in southern Mexico
Source: PLoS Negl Trop Dis. 2024 Jul 5;18(7):e0012301. doi: 10.1371/journal.pntd.0012301 (PMC11262687; doi:10.1371/journal.pntd.0012301)
Supplement: S1 Interview summaries — (ZIP) [file pntd.0012301.s002.zip › vasquez-neri-carter_2024_data_files/Interview Summaries/Interview Summaries/Adrian.docx]

Adrian, [locality name redacted to protect confidentiality] mordido 2020, tenía 23 años

(Entrevista con el padre de Adrián) Alrededor de septiembre de 2020, Adrián (hombre 23 años) regresaba caminando del trabajo en la finca cafetalera cerca de [locality name redacted to protect confidentiality] con su familia. Mordido en la pantorrilla por un cantil negro, *Agkistrodon bilineatus*. Lo bajaron en mula, una caminata de 4 horas. A las 2 horas de caminata llegaron a Potrero Cerro y alguien del pueblo le dio una bebida mezclada de planta de Viborina. Su familia dijo que la Lengua de Suegra lo ayudó mucho. Después de otras 2 horas, Adrián llegó a [locality name redacted to protect confidentiality]. Una vez en la ciudad, la familia llevó a su hijo a [locality name redacted to protect confidentiality] para consultar a un “hierbero”, un familiar lejano. El padre de Adrian sospecha que su hijo se murió porque el doctor le puso “suero,” solución salina, o el hijo bebió agua. La familia dijo que por eso su condición empeoró (se cree que el agua y la solución salina son malas para tratar las mordeduras de serpientes, por sus propiedades frías). Adrian estaba siguiendo las recomendaciones de dieta del hierbero. Adrian comenzó a sangrar por los ojos y los dientes. Murió 4 días después de la mordedura.

“Lo bajamos (a mi hijo, el víctima) como pudimos. Le dieron un licuado de una planta, no me acuerdo cómo fue.”

“Un señor, no se si tiene conocimiento. Era un doctor particular que siempre andaba por aquí, es familiar de mi prima. Ya falleció [el doctor].”

“Lo atendio a mi hijo, ya salió y ya estaba fuera del peligro. Pensamos que estaba salvado, y que se habia administrado bien los medicamentos. No fue al hospital… Tal vez ocupaba recuperar más. Fue rápido (su muerte), supongo que (el doctor) le puso suero, o tomo agua. Si toman agua, es rápida la muerte, dicen, de la culebra… solamente Dios sabe.”

“Era uno de estos hierberos.. no era uno de estos curanderos, daba hierbitas pero daba medicina de potencia. Conocía bastante, fue muy buscado por la gente.”
