# Supplementary material for: Therapeutic itineraries of snakebite victims and antivenom access in southern Mexico
Source: PLoS Negl Trop Dis. 2024 Jul 5;18(7):e0012301. doi: 10.1371/journal.pntd.0012301 (PMC11262687; doi:10.1371/journal.pntd.0012301)
Supplement: S1 Interview summaries — (ZIP) [file pntd.0012301.s002.zip › vasquez-neri-carter_2024_data_files/Interview Summaries/Interview Summaries/Agustin.docx]

Agustin, [locality name redacted to protect confidentiality], mordido 2021 y 2022, tenía 63 y 64 años

Agustín estaba cuidando cafetos en 2021, en enero, cuando una “cotorrera”, *Bothriechis bilineatus*, lo mordió en el dedo de la mano derecha. No tomó ningún medicamento y el hospital más cercano estaba a 9 horas de distancia. Tomó la piel de la serpiente muerta y se la puso en el dedo donde había sido mordido. La mano de Agustín estaba hinchada. Al día siguiente la hinchazón disminuyó. Licuaba y bebía Lengua de Suegra, una planta similar al aloe.

“¿Cómo podemos ir al hospital si no hay nada ahí? Todo cuesta dinero. Si es una serpiente grande, la gente morirá antes de llegar al hospital, así que es mejor quedarse y no moverse… El chile y el alcohol son las mejores curas para neutralizar el veneno. Esto es lo que salva la vida de la gente aquí porque no hay tiempo para llegar hasta el hospital”.

Agustín fue mordido nuevamente en 2022. Se había agachado para desmalezar los cafetos y una pequeña “nauyaca de frío”, Cerrophion godmani, lo mordió en la mano. Esta vez no experimentó dolor ni hinchazón y no utilizó ningún remedio natural.
