# Supplementary material for: Therapeutic itineraries of snakebite victims and antivenom access in southern Mexico
Source: PLoS Negl Trop Dis. 2024 Jul 5;18(7):e0012301. doi: 10.1371/journal.pntd.0012301 (PMC11262687; doi:10.1371/journal.pntd.0012301)
Supplement: S1 Interview summaries — (ZIP) [file pntd.0012301.s002.zip › vasquez-neri-carter_2024_data_files/Interview Summaries/Interview Summaries/Alejandro.docx]

Alejandro, [locality name redacted to protect confidentiality], mordido 2000, tenía 30 anos

En el año 2000, Juan tenía 30 años y fue mordido por primera vez. Estaba cuidando la planta de café cuando le mordió una nauyaca (*Bothrops asper*) en el dedo índice. No pudieron ver la serpiente. La abuela vivía cerca y conoce la medicina natural y qué hacer en caso de una mordedura de serpiente. Compró un litro de alcohol y puré de cedrón (fruta). Juan vomitaba sangre y se le hinchó todo el brazo. Su perro también murió por una mordedura de cantil nauyaca. En aquellos días no había ningún hospital. Ella dice que a las 24 horas después de la picadura de cantil, las personas mueren o se recuperan.

“Ese no se ve en la mata, es color cafecito igual que una hoja. Mi abuela vivía cerca, ella le daba un litro de trago y un pedacito de cedrón. Lo raspaba y lo echaba en el trago. Pero vomitaba mucha sangre de su boca.”

“El nunca fue al hospital. Como mi abuela sabía de medicina, él nunca se fue.”

“Vomitaba mucha sangre, una noche completa.”
