# Supplementary material for: Therapeutic itineraries of snakebite victims and antivenom access in southern Mexico
Source: PLoS Negl Trop Dis. 2024 Jul 5;18(7):e0012301. doi: 10.1371/journal.pntd.0012301 (PMC11262687; doi:10.1371/journal.pntd.0012301)
Supplement: S1 Interview summaries — (ZIP) [file pntd.0012301.s002.zip › vasquez-neri-carter_2024_data_files/Interview Summaries/Interview Summaries/Alfonso.docx]

Alfonso, [locality name redacted to protect confidentiality], mordido en 2012, no se sabe su edad

(Esposo de Carla hablo de otra mordedura)

En Junio o Julio de 2012, Alfonso estaba trabajando en una finca de café cerca a [locality name redacted to protect confidentiality], cuando le mordió una cola blanca. El compañero de Alfonso le cortó su herida, mastico ajo y pimienta y le succiono la herida. Uso una semilla para curar su herida y bajar la hinchazón.
