# Supplementary material for: Therapeutic itineraries of snakebite victims and antivenom access in southern Mexico
Source: PLoS Negl Trop Dis. 2024 Jul 5;18(7):e0012301. doi: 10.1371/journal.pntd.0012301 (PMC11262687; doi:10.1371/journal.pntd.0012301)
Supplement: S1 Interview summaries — (ZIP) [file pntd.0012301.s002.zip › vasquez-neri-carter_2024_data_files/Interview Summaries/Interview Summaries/Ana Maria.docx]

Ana Maria, [locality name redacted to protect confidentiality], mordida 2021, tenía 55 anos

Ana Maria estaba caminando para traer agua en 2021 cuando fue mordida por cola blanca (*Agkistrodon bilineatus*; *Bothrops asper*) en su pie, en su espinilla. Ana Maria y su familia traen agua del río, porque no tienen agua corriente. Ella mató a la serpiente y la trajo consigo, caminando 35 minutos hasta su casa en [locality name redacted to protect confidentiality]. Un vecino la llevó directamente al hospital de [locality name redacted to protect confidentiality], a una hora y media en carro. En el hospital de [locality name redacted to protect confidentiality] recibió un vial de antiveneno. Estuvo en el hospital durante 4 días y fue dada de alta con órdenes de evitar dietas grasas y huevos.
