# Supplementary material for: Therapeutic itineraries of snakebite victims and antivenom access in southern Mexico
Source: PLoS Negl Trop Dis. 2024 Jul 5;18(7):e0012301. doi: 10.1371/journal.pntd.0012301 (PMC11262687; doi:10.1371/journal.pntd.0012301)
Supplement: S1 Interview summaries — (ZIP) [file pntd.0012301.s002.zip › vasquez-neri-carter_2024_data_files/Interview Summaries/Interview Summaries/Angel.docx]

Angel, [locality name redacted to protect confidentiality], mordido 1980, tenía 12 años

Don Ángel estaba regando los cafetos con agua de un río cercano, alrededor del mediodía de noviembre de 1980. No vio el *Bothriechis bicolor* o “cotorrera” porque había mucho follaje de los cafetos. Don Ángel caminaba cuando la serpiente lo mordió en la parte superior del pie derecho. Ángel intentó quitárselo, pero la serpiente se quedó adherida. Ángel corrió a su casa. Cuando la serpiente finalmente se soltó, el padre de Ángel cortó la herida con un cuchillo, entre las dos marcas de los colmillos. Su padre succionó el veneno y la sangre. El padre empezó a sentir mareos y náuseas, pero la sensación pasó. El padre de Ángel empezó a masticar el tabaco de sus cigarrillos y a poner el cataplasma de tabaco en la herida. 20 o 30 minutos después, el padre de Ángel le hizo un torniquete alrededor de la pierna de Ángel, que estaba muy hinchada. El padre de Ángel cargó a Ángel durante 1,5 km para llegar a su casa. En casa, los padres de Ángel le preparaban curarina machacada en tequila. Ángel tenía mucho dolor en su pie y pierna, entonces su madre le vertió agua hirviendo sobre la herida. Dice Ángel que no podía sentir el dolor del agua hirviendo, sólo el de la mordedura de serpiente. El padre de Ángel fue a la casa de otro trabajador a buscar otra cura a base de hierbas, Nauyacol. En los días posteriores a la mordedura de serpiente, Ángel tomaba tragos diarios de curarina en tequila. Durante los dos meses posteriores al accidente, siguió una dieta especial compuesta principalmente de tostadas y café negro.
