# Supplementary material for: Therapeutic itineraries of snakebite victims and antivenom access in southern Mexico
Source: PLoS Negl Trop Dis. 2024 Jul 5;18(7):e0012301. doi: 10.1371/journal.pntd.0012301 (PMC11262687; doi:10.1371/journal.pntd.0012301)
Supplement: S1 Interview summaries — (ZIP) [file pntd.0012301.s002.zip › vasquez-neri-carter_2024_data_files/Interview Summaries/Interview Summaries/Antonio.docx]

Antonio, [locality name redacted to protect confidentiality], mordido 2008, 2010 y 2013, tenía 48, 50 y 53 años

Antonio fue mordido 3 veces.

Primera mordida en 2008, Antonio trabajaba en la plantación de hule cuando lo mordió una sorda, *Bothrops asper*, en la rodilla izquierda. El hermano de Antonio estaba trabajando con él y le dijo que era mejor no caminar para que la sangre no circulara tan rápido con el veneno. Estaban a 15 minutos de su casa y el hermano llevó a Antonio a casa. Cuando llegaron a casa, la visión de Antonio estaba borrosa. Antonio y su hermano le pagaron al vecino 50 pesos para que los llevara al hospital de [locality name redacted to protect confidentiality] (a 15 minutos en auto), donde le administraron un vial de antiveneno. Después de unas 2 o 3 horas en el hospital, la familia de Antonio lo sacó del hospital y lo llevó a Don Pepe, un curandero en [locality name redacted to protect confidentiality]. El curandero le dio a Antonio una bebida de hierbas amargas. Don Pepe pinchó la herida con una aguja, luego quemó alcohol en un frasco de mayonesa limpio para crear un vacío y colocó el frasco sobre la herida para extraer la sangre/el veneno. Don Pepe daba baños de vapor elevando la pierna con un tronco, colocando una toalla mojada sobre la pierna de Antonio y piedras calientes debajo del tronco y la toalla. El curandero oró/cantaba y declaró curada la herida. Otro médico independiente trabajó junto a Don Pepe en [locality name redacted to protect confidentiality], y este médico le inyectaba vitaminas. Don Pepe trató la herida diariamente con bebida de hierbas, ventosa, baños de vapor y oraciones durante 8 días, después de los cuales Antonio se recuperó lo suficiente como para regresar a casa. Don Pepe cobró 600 pesos, sin incluir comidas. Antonio siguió una dieta estricta sin grasas ni carne durante 6 meses y estuvo 6 meses sin poder trabajar.

Antonio estaba nuevamente trabajando en la plantación de hule en 2010, dos años después de la primera mordedura, cuando sufrió otra mordedura de serpiente. Esta vez, una sorda le mordió en la espinilla derecha. El hermano de Antonio, que estaba con él, lo llevó 15 minutos hasta su casa. El vecino los llevó nuevamente al hospital de [locality name redacted to protect confidentiality], pero esta vez no le cobró a Antonio porque estaban trabajando en la plantación de caucho del vecino cuando ocurrió la segunda mordida. Llegaron 30 minutos después de la picadura, pero el hospital no tenía antídoto. Antonio acudió a la casa de Don Pepe donde fue tratado con bebida de hierbas, ventosa, baños de vapor y oraciones diarias durante un mes. Don Pepe cobraba 800 por sus servicios, sin incluir comidas. Antonio tuvo que seguir una estricta dieta libre de grasas (compuesta principalmente de tortillas secas y caldo de pollo) durante 6 meses, y permaneció en casa durante un año sin trabajar.

Tres años después de la segunda mordedura, Antonio fue mordido por tercera vez en la finca de caucho. Un robahueso lo mordió en la rodilla y Antonio se dirigió directamente a la casa de Don Pepe en [locality name redacted to protect confidentiality], a unos 30 minutos en auto. Allí permaneció dos meses, recibiendo tratamiento con bebida de hierbas, ventosa, baños de vapor y oraciones diarias. Sangraba por los ojos, los oídos y los dientes, y se le cayeron dos dientes. El médico que trabajaba junto a Don Pepe hacía controles periódicos de la sangre y los ojos de Antonio. Don Pepe quería que Antonio aprendiera el tratamiento para las mordeduras de serpiente en caso de que ocurriera otro accidente. El curandero llevó a Antonio a la montaña para ver las hierbas, pero Antonio no recuerda el nombre. Dijo que la hierba era una enredadera con hojas largas en forma de corazón. Antonio ya no quiere trabajar en el campo porque lo siguen picando. La pierna de Antonio perdió algo de músculo y hay una hendidura en la pantorrilla donde ocurrió la mordida.

“Me mordió, y andaba mi hermano conmigo. El veneno corre si uno camina, entonces mi hermano no me dejó caminar, me trajo cargando de ahí. Ya me estaba nublando la vista. Lo bueno es que fui al doctor enseguida y ya me pusieron esa medicina contra el veneno. Me quedé 2-3 horas en el hospital mientras me pusieron el antiveneno. No alcance a ver cuantos frascos me pusieron. Ya no me dejaron ahí, mi familia, me sacaron [del hospital] y me dejaron con el culebrero. Estuve en la casa de Don Pepe por 8 días. Me dio una toma amarga, yo vi que raspaba un tipo hueso, lo hizo polvo. Y me daba en vaso. El señor me sacaba el veneno, y estaba trabajando con otro doctor que me puso vitamina. Me quemó la sangre el veneno de la víbora, entonces me pusieron vitamina. Le puso un vaso y saco liquido amarillo amarillo. Me hizo baño de vapor. Me puso mi pie arriba de un tronco, lo tapó bien, calentaba una piedra y con el vapor lo hacía sudar donde picó la culebra. Me hizo oración cuando acababa de curar, pero ya se me olvido. Después de eso volví a mi casa pero no trabaje enseguida, demore. No trabajé unos 6 meses y de ahí volví a trabajar en el campo.”

“Las tres veces estuve con mi hermano. Fui al hospital la segunda vez, pero ya no tenían. Buscaron, buscaron pero no había. Nos demoramos… Mi familia me sacó y me llevó a Don Pepe.”

“Hay que pagar [el traslado].”

“En la segunda vez me quedé con Don Pepe un mes. La toma era todos los días, y el doctor todos los días. Me cobró 800 pesos. Me quedé en mi casa un año sin trabajar después de la segunda vez. Te ponen mucha dieta. Lo único que puedes comer es pan tostado. Nada de comida. No hay dinero a veces, entonces comimos tortilla seca o caldo de pollo sin carne, puro caldo. Se evita la grasa por el veneno. Y si lo vas a comer, asado. Estuve con la dieta unos 6 meses. Hice la dieta 3 veces.”

“La tercera vez estuvo más peligroso porque estuve casi 3 meses internado con Don Pepe. Me saco sangre por todos los poros hasta el oído. La tercera vez fui directamente con Don Pepe. Como este señor trabajaba junto con el doctor particular. Todos los días checo la vista y la sangre. No se como pero me dijo que el mismo veneno convierte la sangre en agua.”

“Esta tercera vez, Don Pepe ya quería que yo aprendiera a curar. Me llevaba al cerro, me mostraba las hierbas. Pero ya no me acuerdo. Era un bejuco con hojas largas. Dijo su oración tres veces, muy seguido. Me dice, ‘tomate unos 3 limones y tomatelos aunque esté agrio para que te corte el veneno en seguida. Ya con eso vas tranquilo a tu casa.”

“Me siento mejor con Don Pepe porque saca el veneno y es más rápido. Mi hijo estuvo 3 meses. Los médicos no lo cortan, pero Don Pepe lo rajo. Antes de hacer la ventosa lo picaba. El médico ponía anestesia y lo cortaba y no había nada. El analizaba si se ponía negro por dentro.”
