# Supplementary material for: Therapeutic itineraries of snakebite victims and antivenom access in southern Mexico
Source: PLoS Negl Trop Dis. 2024 Jul 5;18(7):e0012301. doi: 10.1371/journal.pntd.0012301 (PMC11262687; doi:10.1371/journal.pntd.0012301)
Supplement: S1 Interview summaries — (ZIP) [file pntd.0012301.s002.zip › vasquez-neri-carter_2024_data_files/Interview Summaries/Interview Summaries/Arturo.docx]

Arturo, [locality name redacted to protect confidentiality], mordido 1983, tenía 15 años

Arturo trabajaba en el cafetal en 1983, cuando tenía 15 años. Arturo intentó mover una rama de café hacia un lado cuando el Bothriechis bicolor le mordió dos veces la mano. Arturo después vio los 4 huecos de los colmillos. La serpiente era de color verde brillante con el vientre pálido. Arturo le puso gasolina diesel en la herida. Trituró curarina y le añadió alcohol. Arturo no fue al hospital porque no había clínica ni hospital cerca. Su brazo estuvo hinchado durante 40 días. Arturo se siente más cómodo en el hospital, pero usaba hierbas porque en esos tiempos no había ningún centro de salud cerca de él.

“No lo saque [al veneno], lo que hice fue embarrar con diesel. Hay personas que se les muerde esa culebra, la cotorrera verde y no se hinchan. Yo me hinché, me hincharon mis piernas, mis brazos. Por poco me moría yo.”

“Lo cure un un monte que lo llaman curarina. Lo tomé machucadito con alcohol. En este tiempo no había clínica, no había hospital, no había nada.”

“Si me mordiera ahora una serpiente me iría al municipio al hospital, donde tienen medicina. Ahora hay doctores. Antes, como? Había puro monte. Eso de hierba no basta.”

“La curarina, la viborina creo que lo dicen. Hay muchas personas que les muerde la víbora, pero no se mueren por esas frutitas, lo muelen y así lo toman. Y han logrado con ese, no mueren.”

“Si hay medicamento para la culebra.”

“Hemos aplastado a la víbora con una caña. Y la ye damos que de muerde esa cana. Lo hemos visto que cuando la luna está redonda, es cuando pesa la ponzoña de la culebra. En la luna tiernita, es cuando está clarita el veneno.”
