# Supplementary material for: Therapeutic itineraries of snakebite victims and antivenom access in southern Mexico
Source: PLoS Negl Trop Dis. 2024 Jul 5;18(7):e0012301. doi: 10.1371/journal.pntd.0012301 (PMC11262687; doi:10.1371/journal.pntd.0012301)
Supplement: S1 Interview summaries — (ZIP) [file pntd.0012301.s002.zip › vasquez-neri-carter_2024_data_files/Interview Summaries/Interview Summaries/Carla.docx]

Carla, [locality name redacted to protect confidentiality], Mordida 1988, tenía 10 años

Carla, mujer Tzotzil de 10 años, estaba cuidando los guajolotes de su familia, cerca al pozo cuando una “basurera” no venenosa (*Drymarchon melanurus*) le mordió la rodilla en 1988. Carla regresó corriendo a la casa con la serpiente todavía pegada a su rodilla hasta que el padre lo logró sacar. La familia de Carla cortó la picadura con fragmentos de vidrio, succionó la sangre y aplicó una cataplasma de puré de ajo en la herida. También le pusieron una semilla parecida al algodón que reduce la inflamación (no recordaban el nombre). Sus padres le dieron “caña”, un licor de caña de azúcar, y la llevaron a un curandero. El curandero felicitó a la familia por el buen trato y recomendó sólo otra taza de caña. Cuando se le preguntó si prefiere hospitales o “medicina natural”, Carla mencionó que en esos días no había ningún hospital cerca. Ahora no tiene cicatriz o secuela de su accidente.

“El mejor remedio es la caña.”

“Fue una basurera que mordió a mi esposa, de los que andan en el arroyo. Uno verdecito.”

“Me llevaron al doctor pero no me hizo nada porque mi papá me picó toda mi herida y me chupo la sangre. Chupo con ajo, mastico el ajo y lo chupo. Y el doctor (curandero) dijo, ‘ya hiciste bueno, solo le das una copita de caña.’ Me sané con eso.”

“En ese tiempo no había clinica o hospital cerca. Solo había Don Lucio (el curandero). El ya se murió”

“Dicen (que hay secuelas) cuando no sale todo el veneno. Pero mi papá lo chupo todo, no quedó veneno creo.”
