# Supplementary material for: Therapeutic itineraries of snakebite victims and antivenom access in southern Mexico
Source: PLoS Negl Trop Dis. 2024 Jul 5;18(7):e0012301. doi: 10.1371/journal.pntd.0012301 (PMC11262687; doi:10.1371/journal.pntd.0012301)
Supplement: S1 Interview summaries — (ZIP) [file pntd.0012301.s002.zip › vasquez-neri-carter_2024_data_files/Interview Summaries/Interview Summaries/Carlos (Padre de Sebastian).docx]

Carlos, [locality name redacted to protect confidentiality]

El padre de Sebastián (Carlos) también fue mordido en 2021 o 2022 (no recuerda exactamente cuándo) y tomó curarina con aguardiente. Al día siguiente fue al médico y el médico le dijo que después de 24 horas no había nada que hacer. Otros remedios caseros los había aprendido de otras familias en el hospital cuando estaba con su hijo en el hospital de [locality name redacted to protect confidentiality]. Recomendaron “tripa de calabaza” mezclada y otras hierbas. Los padres de Sebastián creen que es útil conocer estas plantas porque no tienen clínica en [locality name redacted to protect confidentiality] y necesitan sobrevivir el viaje al hospital.

“La curarina es la raíz de un bejuco. Lo arranque, lo lave y lo machuque. Estaba al lado del río, tomaba agua. El otro dia fui al doctor, pero me dijo ‘no, ya han pasado más de 24 horas. Ya no hay nada.’ No me hizo nada. Pero cuando estaba en [locality name redacted to protect confidentiality] [por la mordida de su hijo] hablé con muchas personas, y me dieron ideas de muchos árboles para la hora que no hay medicina. Una muchacha me receto, me dijo que la tripa de calabaza también es buena. esto es otro.”

“En lo que corre uno a trasladarlo al doctor, se pone remedios caseros.”
