# Supplementary material for: Therapeutic itineraries of snakebite victims and antivenom access in southern Mexico
Source: PLoS Negl Trop Dis. 2024 Jul 5;18(7):e0012301. doi: 10.1371/journal.pntd.0012301 (PMC11262687; doi:10.1371/journal.pntd.0012301)
Supplement: S1 Interview summaries — (ZIP) [file pntd.0012301.s002.zip › vasquez-neri-carter_2024_data_files/Interview Summaries/Interview Summaries/Christofer.docx]

Christofer, [locality name redacted to protect confidentiality], mordido 2013, tenía 41 años

Christofer, hombre Tzotzil de [locality name redacted to protect confidentiality], estaba cortando café en Marzo de 2013 cuando lo mordió una cotorrera, ambas bicolores en el dedo. Intentó exprimir la sangre. No tuvo síntomas porque come mucho chile.

“Nosotros, en aquel tiempo no sabíamos si había remedio o pastilla o inyección. Como nosotros acostumbramos tomar agua de chile, una tasa lo batimos y con eso se quitó el dolor. Tomemos posh al último.”

“Como somos campesinos, no sabemos cómo curar. ¡Duele mucho! Había hospital pero hasta allá, lejos.”

“Nosotros comemos bastante chile. Una vez me pico uno de estas serpientes verdes, me pico en mi dedo. No se hincho. No se hincho porque comemos mucho chile. Pero cuando no come chile uno, pues ayy noo. Puta, el dolor! Pero para mí, no se hincho nada.”
