# Supplementary material for: Therapeutic itineraries of snakebite victims and antivenom access in southern Mexico
Source: PLoS Negl Trop Dis. 2024 Jul 5;18(7):e0012301. doi: 10.1371/journal.pntd.0012301 (PMC11262687; doi:10.1371/journal.pntd.0012301)
Supplement: S1 Interview summaries — (ZIP) [file pntd.0012301.s002.zip › vasquez-neri-carter_2024_data_files/Interview Summaries/Interview Summaries/Claudia.docx]

Claudia, [locality name redacted to protect confidentiality], mordida 2003, no de sabe cuantos anos tenia

(Hija de Claudia hablando) fue mordida cuando subía la montaña hacia su casa en 2003. La mordieron en la pantorrilla. Su pierna se hinchó y cuando llegó a casa no podía caminar. A veces había brigadas de salud en el pueblo, y por suerte estaban allí en ese momento. El yerno salió a traer antídotos de la brigada, porque sabía que normalmente los llevan consigo. El yerno inyectó a la víctima aproximadamente una hora y media después de la mordedura. No usaron hierbas ni le dieron alcohol.

“Antes venían esos doctores de filaria. Ellos por suerte estaban aquí, y traían la inyección para mordedura de culebra. Se fue mi marido para traerlo y lo inyectó.
