# Supplementary material for: Therapeutic itineraries of snakebite victims and antivenom access in southern Mexico
Source: PLoS Negl Trop Dis. 2024 Jul 5;18(7):e0012301. doi: 10.1371/journal.pntd.0012301 (PMC11262687; doi:10.1371/journal.pntd.0012301)
Supplement: S1 Interview summaries — (ZIP) [file pntd.0012301.s002.zip › vasquez-neri-carter_2024_data_files/Interview Summaries/Interview Summaries/Cristina.docx]

Cristina, [locality name redacted to protect confidentiality], mordida 2008, tenía 25 años

(Sobrina de Cristina) Cristina estaba limpiando café en 2008 cuando fue mordida por un cantil, *Agkistrodon bilineatus*, en el brazo. Fue a casa de su abuela porque la abuela conoce los remedios tradicionales. Cortaron la herida e intentaron succionar el veneno. Ella no sobrevivió al viaje al hospital.
