# Supplementary material for: Therapeutic itineraries of snakebite victims and antivenom access in southern Mexico
Source: PLoS Negl Trop Dis. 2024 Jul 5;18(7):e0012301. doi: 10.1371/journal.pntd.0012301 (PMC11262687; doi:10.1371/journal.pntd.0012301)
Supplement: S1 Interview summaries — (ZIP) [file pntd.0012301.s002.zip › vasquez-neri-carter_2024_data_files/Interview Summaries/Interview Summaries/Daniela.docx]

Daniela, [locality name redacted to protect confidentiality], mordida 2013, tenía 22 años

Daniela tenía 22 años, estaba cuidando plantaciones de judías verdes y maíz en la montaña cerca de [locality name redacted to protect confidentiality] en 2013. Sintió un mordisco en el dedo del pie, pero al principio pensó que era una piquete hormiga. Solo vio un agujero de mordida, por lo que sospecha que sus sandalias detuvieron el otro colmillo. Ella nunca vio a la serpiente. Cuando llegó a casa, tenía el pie y la pierna hinchada y sentía fiebre y dolor en los huesos. Fue al hospital de [locality name redacted to protect confidentiality] y el médico le dijo que lo más probable era una picadura de araña y la mandó a su casa. Esa noche se fue a dormir, pero el domingo por la mañana, cuando se despertó, Daniela encontró sangre de la boca en la almohada. Fue al hospital de [locality name redacted to protect confidentiality], donde pasó la noche, pero el laboratorio estaba cerrado (siempre están cerrados los domingos). El lunes le realizaron análisis de sangre y descubrieron que se trataba de una mordedura de serpiente. No tenían el antídoto en [locality name redacted to protect confidentiality], entonces la derivaron a [locality name redacted to protect confidentiality], a una hora de distancia. “mínimo uno tiene que tener dinero porque sino no te van a atender” (hablando de hospitales de [locality name redacted to protect confidentiality]). Luego la trajeron a [locality name redacted to protect confidentiality], pero no quisieron atenderla porque habían pasado tres días de la mordedura.

Ella cree que su pie no se puso negro (necrosis) porque la serpiente no le metió ambos colmillos.

“Se murió otro chavo aquí que sembró jengibre y le picó una víbora en el brazo. Ya no logro bajar. Tenía como 32 años y dejó como 3 niños a su esposa. Murió muy mal. Habia otra nino de 12 anos en [locality name redacted to protect confidentiality] que estaba pescando y le mordio la vibora, y le puso negro todo su pierna. Estuvo internado 3 meses en [locality name redacted to protect confidentiality]. Dicen que el veneno lo carcomió, quedó hueso hueso. Camina mal. Pensamos que ya no iba a sobrar la pierna. Por eso en cada pueblo deben de tener el antídoto. Aquí hay bosque, hay monte. No es como la ciudad, no hay serpientes en la ciudad. El gobierno se queda con los recursos que deben de ser para el pueblo.”

“No más me sentí un piquete. Se me hizo una bola. Llegué a casa y me bañé, pero se me empezó a hinchar. Pero nunca se me hizo negro porque no me inyecto todo el veneno, porque no puso los dos colmillos, supongo. Me internaron en [locality name redacted to protect confidentiality], pero no tenían el antídoto. Me bajaron a [locality name redacted to protect confidentiality] pero no me querían atender. Dijeron que ya había pasado mucho tiempo la víbora. Pero me sangraban todos los dientes, sentí que me iba a morir.”

“Yo pienso que deberían de tener el antídoto en todas las clínicas, como aquí el centro de salud es muy chiquitito, nosotros no tenemos doctores. Cuando es muy grave vamos a [locality name redacted to protect confidentiality] o [locality name redacted to protect confidentiality]. Y minimo tienes que tener dinero porque sino no te van a atender. El antídoto de la víbora es caro.”

“Aquí en este pueblo deben de tener el antídoto, porque imagínate de aquí a [locality name redacted to protect confidentiality] o [locality name redacted to protect confidentiality], ya te pasa el veneno. Aquí lo que hacen la gente, te amarraran pero te pasa en veneno por la sangre.”

“Cuando muerde la vibora, se te pone negro en seguida.’
