# Supplementary material for: Therapeutic itineraries of snakebite victims and antivenom access in southern Mexico
Source: PLoS Negl Trop Dis. 2024 Jul 5;18(7):e0012301. doi: 10.1371/journal.pntd.0012301 (PMC11262687; doi:10.1371/journal.pntd.0012301)
Supplement: S1 Interview summaries — (ZIP) [file pntd.0012301.s002.zip › vasquez-neri-carter_2024_data_files/Interview Summaries/Interview Summaries/Edgar.docx]

Edgar, [locality name redacted to protect confidentiality], mordido en 2022, tenía 36 años

Edgar estaba trabajando en el cafetal el 21 de noviembre de 2022 cuando puso su mano cerca del suelo y fue mordido en la mano por un “cantil”, *Agkistrodon bilineatus*. Edgar caminó hasta su casa, una caminata de 15 minutos, donde su madre le dio curarina (una enredadera, un bejuco) con aguardiente. Luego de esto, se dirigieron inmediatamente a Compañeros de Salud, a 5 minutos de su domicilio. No tenían antídoto en existencia, por lo que Edgar viajó 40 minutos en motocicleta hasta [locality name redacted to protect confidentiality], donde compró un frasco de antídoto a una enfermera por 3.000 pesos. La enfermera le inyectó el antídoto por vía intramuscular y le recomendó a Edgar ir al hospital de [locality name redacted to protect confidentiality], porque necesitaba más antídoto pero era caro. Edgar estuvo en Paraíso unos 30 minutos antes de partir hacia [locality name redacted to protect confidentiality]. Desde [locality name redacted to protect confidentiality] hasta [locality name redacted to protect confidentiality] son aproximadamente 2,5 horas. En el hospital recibió un total de 16 viales de antídoto. Edgar dice que todavía le duele la mano.

“Vine para ver si tenían medicamento ahí en la clínica, allá en el centro. Ahí estaban ellos pero no tenían medicamentos.”

“Era bastante evidente que si inyecto veneno. De aquí para allá, estaba sangrando mi boca.”
