# Supplementary material for: Therapeutic itineraries of snakebite victims and antivenom access in southern Mexico
Source: PLoS Negl Trop Dis. 2024 Jul 5;18(7):e0012301. doi: 10.1371/journal.pntd.0012301 (PMC11262687; doi:10.1371/journal.pntd.0012301)
Supplement: S1 Interview summaries — (ZIP) [file pntd.0012301.s002.zip › vasquez-neri-carter_2024_data_files/Interview Summaries/Interview Summaries/Eduardo.docx]

Eduardo, [locality name redacted to protect confidentiality], mordido en 2019, tenía 45 años

Eduardo, hombre de 45 años, estaba trabajando en el cafetal en 2019, cuando fue mordido por un cantil, *Agkistrodon bilineatus*, en la muñeca. Un compañero de trabajo le ató un torniquete en el brazo. El sobrino lo llevó a [locality name redacted to protect confidentiality], donde le quitaron el torniquete y (la víctima) empezó a tener problemas para respirar. No había ningún antídoto. Inmediatamente lo derivaron a [locality name redacted to protect confidentiality], pero allí tampoco tenían antídoto. Un médico sugirió que el siguiente paso sería [locality name redacted to protect confidentiality], pero cuando llegara ya estaría muerto. Tenía algunos familiares que buscaron por todos lados, y finalmente encontraron antídotos en el centro veterinario. Compraron 5 viales a 3500 pesos cada uno. Tuvieron que pagar 27.000 pesos en total por el transporte, ambulancia, medicinas y todo.

“Corremos riesgos nosotros aquí en lo que es el campo, pues. Y a varios les han mordido las serpientes. Nos hemos ayudado como hemos podido, nos integramos en diferentes maneras por no haber recursos. Lo que queremos es salvarnos pues. Nosotros estamos retirados de lo que es [locality name redacted to protect confidentiality]. este hombre fue mordido por una serpiente, y vino mal. Y tuvimos que acercarnos y ver la manera de cómo dar una medicina casera ahí para poder entretener el veneno, porque eso ya penetra. Yo cuando me mordió, estaba limpiando café. Mi sobrino me hizo el favor de llevarme. Llegando a [locality name redacted to protect confidentiality], no había medicina, nada. Ahí se me quiso tapar la respiración. Me trasladaron inmediatamente a [locality name redacted to protect confidentiality]. Llegando allá, que no había medicamento allí tampoco. Me hablaron que consiguieron uno en [locality name redacted to protect confidentiality], pero me dijo la doctora ‘aquí ya que venga el medicamento, tú ya estás muerto.’ Pues tuvimos que buscarle ahí, se fue mi familia a un veterinario de animales. Ahí es donde conseguimos la ampolleta, pero son caras. 3,500 pesos la ampolleta, y me pusieron como 5. Gastamos unos 27 mil pesos con todo, con el traslado de aquí. Sería muy útil tener un medicamento aquí, estamos en riesgo aquí en el campo. No hay manera de salvarnos. En tiempo de lluvia es cuando hay más riesgo, [las serpientes] empiezan a producirse más.”

“Desgraciadamente no había nada en el hospital, ni medicamento, nada. Llegue al otro hospital, y tampoco.”

[Mujer a su lado, vecina] “Ya lo lloramos, se puso bien grave”

“Gracias a dios lo solucionamos pero tenemos que endeudarnos para poder sobrevivir a esto. Nos apoyamos cuando nos tenemos que trasladar. lo que queremos es salvarnos pues.”
