# Supplementary material for: Therapeutic itineraries of snakebite victims and antivenom access in southern Mexico
Source: PLoS Negl Trop Dis. 2024 Jul 5;18(7):e0012301. doi: 10.1371/journal.pntd.0012301 (PMC11262687; doi:10.1371/journal.pntd.0012301)
Supplement: S1 Interview summaries — (ZIP) [file pntd.0012301.s002.zip › vasquez-neri-carter_2024_data_files/Interview Summaries/Interview Summaries/Emilio.docx]

Emilio, [locality name redacted to protect confidentiality], mordido 2016, tenía 40

Emilio tenía 40 años, estaba trabajando en [ranch name redacted to protect confidentiality]. Estaba limpiando las plantas de café alrededor de las 10 de la mañana de julio de 2016 cuando lo mordió un “cantil color basura, no la de agua”, (tal vez *Cerrophion godmani*). en la mano. Rápidamente caminó 10 minutos hasta el centro agrícola, donde su jefe le hizo un torniquete. Unos 20 minutos después de la mordedura, su jefe le dio curarina (una hierba) en trago y nauyacol (un jarabe). Emilio vomitó la medicina. Condujo la camioneta familiar durante 20 minutos para llegar a su casa en la parte baja de [locality name redacted to protect confidentiality]. Luego, acudió a la clínica de [locality name redacted to protect confidentiality]. La enfermera le inyectó un antídoto por vía intramuscular, independientemente de la clínica. Como el antiveneno era caro, Emilio solo tomó uno y fue al hospital de [locality name redacted to protect confidentiality] (1,5 h). No tenían antiveneno, así que fueron a [locality name redacted to protect confidentiality] (2,5 horas) donde le inyectaron 3 frascos más. Después de cuatro días salió del hospital y todavía tuvo dolores durante unos seis días.

“Lo más inmediato, lo llevamos a la enfermera aquí y le inyectaron un frasquito. Pero es caro… Le pusimos un frasco aquí para que aguante hasta [locality name redacted to protect confidentiality], y de [locality name redacted to protect confidentiality] a [locality name redacted to protect confidentiality] porque no le aceptaron en [locality name redacted to protect confidentiality].”

(Esposa de Emilio) “[Emilio] dice que todavía se le acalambra su mano.”
