# Supplementary material for: Therapeutic itineraries of snakebite victims and antivenom access in southern Mexico
Source: PLoS Negl Trop Dis. 2024 Jul 5;18(7):e0012301. doi: 10.1371/journal.pntd.0012301 (PMC11262687; doi:10.1371/journal.pntd.0012301)
Supplement: S1 Interview summaries — (ZIP) [file pntd.0012301.s002.zip › vasquez-neri-carter_2024_data_files/Interview Summaries/Interview Summaries/Enrique.docx]

Enrique, [locality name redacted to protect confidentiality], mordido 2022, tenia 15 anos

(Entrevista al jefe de Enrique) En julio de 2022, un niño indígena de la montaña de 15 años llamado Enrique estaba trabajando en una finca cafetera. Fue a revisar la manguera y una serpiente de cascabel, *Crotalus culminatus*, le mordió en la pantorrilla. Hay pastos altos y arbustos alrededor de esa zona. Inmediatamente se dirigió a la casa del rancho (15 minutos caminando), donde su jefe le hizo un torniquete y le dio alcohol con viborina. Enrique sangraba y sentía dolor. Enrique pagó un taxi para llegar a [locality name redacted to protect confidentiality], un pueblo más grande. Estuvo allí 8 días. Pasó más o menos un mes antes de que Enrique se recuperara.

“Aquí nosotros usamos una planta que se llama Viborina. Es amarga, amarga, una enredadera. Lo machacamos y lo ponemos en el trago. Y por un piquete de alacrán o serpiente, lo usamos. Hay un jarabe también, y dicen que es bueno. Se llama el Nauyacol… Se le pican, lo rajan, lo benden con torniquete para que se aguante hasta el hospital, porque es de aquí hasta [locality name redacted to protect confidentiality].”

“Bajo un chamaco que se le había picado y se lo di [la viborina]. El niño era indigena de aquí de la sierra. No se si le cayo bien… Se hinchó su pie y estaba sangrando la herida. Lo llevaron a [locality name redacted to protect confidentiality]. Jaló un taxi para [locality name redacted to protect confidentiality] y pasó 8 días allá. Y cuando regresó le dolía mucho.”

“Cuando se le muerde una culebra no hay que mojarse, no, es malo. Dicen, pues.”

“Normalmente de aquí a [locality name redacted to protect confidentiality] cobran como 150 pesos, pero como es especial, ahí cobran más. No sé cuánto le cobraron a este muchacho el taxi.”
