# Supplementary material for: Therapeutic itineraries of snakebite victims and antivenom access in southern Mexico
Source: PLoS Negl Trop Dis. 2024 Jul 5;18(7):e0012301. doi: 10.1371/journal.pntd.0012301 (PMC11262687; doi:10.1371/journal.pntd.0012301)
Supplement: S1 Interview summaries — (ZIP) [file pntd.0012301.s002.zip › vasquez-neri-carter_2024_data_files/Interview Summaries/Interview Summaries/Eugenia.docx]

Eugenia, [locality name redacted to protect confidentiality], mordida 1980, tenía 20 años

Eugenia fue mordida el 8 de diciembre de 1980 a las 6 de la mañana. Estaba caminando para ayudar a su hermana con un trabajo cuando un cantil nauyaca (*Agkistrodon bilineatus*) la mordió en la espinilla izquierda. Eugenia regresó a su casa y la serpiente la seguía. Fue mordida a unos 50 metros de su casa. Se desmayó en el camino de regreso. Sus hermanas la encontraron y la llevaron adentro de la casa. Su familia le dio ajo y cedrón de comer, y le pusieron alcanfor en la herida. Unos minutos más tarde, le quemaron el lugar de la herida con un machete caliente. Su pierna se hinchó y sintió un dolor ardiente por el veneno. Su familia fue a buscar a un médico local para que llegue a la casa. Un médico en el Ejido [locality name redacted to protect confidentiality] llegó a la casa de Eugenia, y le inyectó un antídoto el día del accidente y otro antídoto al día siguiente. Ella siente que el antídoto no tuvo ningún efecto. Sintió dolores y molestias generales, con dolores de cabeza estos días. Unos días después, su esposo le pagó a otro médico de [locality name redacted to protect confidentiality] para que la atendiera por sus dolores de cabeza. Estuvo en cama 40 días y siguió una dieta especial. No podía comer carne ni grasas.
