# Supplementary material for: Therapeutic itineraries of snakebite victims and antivenom access in southern Mexico
Source: PLoS Negl Trop Dis. 2024 Jul 5;18(7):e0012301. doi: 10.1371/journal.pntd.0012301 (PMC11262687; doi:10.1371/journal.pntd.0012301)
Supplement: S1 Interview summaries — (ZIP) [file pntd.0012301.s002.zip › vasquez-neri-carter_2024_data_files/Interview Summaries/Interview Summaries/Federico.docx]

Federico, [locality name redacted to protect confidentiality], mordido en 2008, tenía 42 años

Federico estaba limpiando su plantación personal de maíz de aproximadamente una hectárea y media en [locality name redacted to protect confidentiality]. El maíz estaba casi maduro y Federico calzaba sandalias. Pasó por un arroyo de agua alrededor de las 9 de la mañana, donde lo mordió una sorda, Bothrops asper, en el pie, y luego la serpiente lo mordió nuevamente alrededor de las 10 de la mañana. Dijo que al principio las picaduras no le dolían mucho. Federico fue primero a un curandero en [locality name redacted to protect confidentiality], pero el curandero no hizo nada más que darle ajo crudo para comer. La pierna de Federico empezó a ponerse morada, por lo que el curandero lo dejó ir sin pagar. Federico fue al centro de salud a las 10:30, y de ahí al hospital de [locality name redacted to protect confidentiality] y llegó alrededor de las 12 del día. Estaban haciéndole pruebas cuando Federico empezó a vomitar sangre. El hermano de Federico habló con las autoridades del hospital para que trasladaran a Federico a [locality name redacted to protect confidentiality], donde tenían antiveneno. Llegaron al hospital de [locality name redacted to protect confidentiality].

Le mordió cerca de un arroyo y el dice que tuvo que pasar el arroyo. Federico dice que cuando te muerde una víbora y toca el agua es malo.

como 10:30 se fue de donde ocurrió la mordedura en pasaje público pero le dijo a una señora de nombre Luisa que le dijera a su hija de esa señora para que esa muchacha avisara a sus hermanos del accidente y primero llegó a las orillas de [locality name redacted to protect confidentiality] con un curandero, y el curandero le dijo que tenían que esperar hasta que su pie se quedara morado. Para este momento su hermano ya había llegado ahí y ambos dijeron que no iban a esperar a que se pusiera morado y decidieron irse al centro de salud de [locality name redacted to protect confidentiality] que ahora es hospital, pero ahí solo le pusieron un suero. Su hermano le pregunto al doctor que si no se iba a empeorar con solo eso, y el doctor dijo que no. El hermano de Federico casi discutía con el doctor justo cuando se estaba acabando el suero, porque Federico empezó a escupir sangre en ese momento. Un presidente en ese tiempo estaba dando su informe de gobierno el hermano de Federico decidió ir a pedirle ayuda al presidente y se tuvo que meter entre todas las personas hasta llegar con el presidente y pedirle ayuda, lo cual el presidente aceptó y cubrió el traslado de [locality name redacted to protect confidentiality] hasta [locality name redacted to protect confidentiality] y cubrió medicamentos, pero les advirtió que no lo atendiera ningún curandero porque si se moría, él no se haría responsable de eso. Entonces la víctima llegó como a la 1 pm y a las 2 le atendieron y fue porque una señora le dio su pase para que ingresara rápido. Federico se sentía demasiado mal dice que ya no veía la luz dice que le pusieron 3 sueros y dice que 1 ampolleta y se quedó internado 3 días y después del 3er día llegó a verlo un curandero y le dio unas hierbas para desinflamar. Se desinflamó a los 8 días después de que le dieron de alta del hospital de [locality name redacted to protect confidentiality].

Dice Federico que cuando hay rayos (tormentas eléctricas) le duele en el sitio

Dice que no existe ningún medicamento para las mordeduras de serpientes coralinas.

“Hay medicamento de la sorda, porque hay que apurarse porque este pica enseguida. Dos piquetes me pasaron

“Mi maíz ya estaba grande estaba en elote. Llegué borracho. Yo no usaba zapatos. No se siente mucho cuando pica. A las 9 me picó la primera vez, y ya me estaba haciendo efecto cuando a las 10 me picó otra vez. Era una sorda. Cuando pica, se hincha de todos lados. Ese centro de salud aquí nos dio paso para [locality name redacted to protect confidentiality]. A las 2 me atendieron allá. Ya no podía caminar.
