# Supplementary material for: Therapeutic itineraries of snakebite victims and antivenom access in southern Mexico
Source: PLoS Negl Trop Dis. 2024 Jul 5;18(7):e0012301. doi: 10.1371/journal.pntd.0012301 (PMC11262687; doi:10.1371/journal.pntd.0012301)
Supplement: S1 Interview summaries — (ZIP) [file pntd.0012301.s002.zip › vasquez-neri-carter_2024_data_files/Interview Summaries/Interview Summaries/Francisco.docx]

Francisco, [locality name redacted to protect confidentiality], mordido 2023, tenía 60 años

Francisco, hombre tsotsil de 60 años, estaba cuidando cafetos en julio de 2023 cuando una “cola blanca” (probablemente *Agkistrodon bilineatus* o *Bothrops asper*) le mordió la mano. Mató a la serpiente, luego licuó chile, se lo bebió y comió ajo crudo. "El veneno de serpiente es frío, por lo que debes beber cosas calientes para curarlo". Su mano no se hinchó, por lo que Francisco cree que la serpiente ya había comido. Cuando la serpiente come, según las creencias locales, ella (la serpiente) no hace daño a nadie.

“Al chile lo bati y me lo tomé. Tenía yo ajo, lo mastique yo bien. Porque el ajo es caliente. En cuestión de la culebra que es frío, hay que tomar cosas calientes. El chile también es bueno porque es caliente. Ya no de hincho porque dicen que ya había comido la culebra. Cuando come la serpiente dicen que no hace nada. Pero cuando no come, si se hincha. Maté a la serpiente.”
