# Supplementary material for: Therapeutic itineraries of snakebite victims and antivenom access in southern Mexico
Source: PLoS Negl Trop Dis. 2024 Jul 5;18(7):e0012301. doi: 10.1371/journal.pntd.0012301 (PMC11262687; doi:10.1371/journal.pntd.0012301)
Supplement: S1 Interview summaries — (ZIP) [file pntd.0012301.s002.zip › vasquez-neri-carter_2024_data_files/Interview Summaries/Interview Summaries/Gabriel.docx]

Gabriel, [farm name redacted to protect confidentiality], mordido en 2023, tenía 23 años

Gabriel, hombre Tsotsil de 23 años, es dueño de la Finca [farm name redacted to protect confidentiality], una finca de café cerca a la reserva del [locality name redacted to protect confidentiality]. Estaba caminando entre los cafetales, checando a los trabajadores cuando fue mordido por una serpiente no venenosa en febrero de 2023. Mató a la serpiente para tomarle fotos. Gabriel marco a un curandero que conoce de su pueblo de [locality name redacted to protect confidentiality]. Pidió a un compañero que le lleve a [locality name redacted to protect confidentiality], donde había este curandero conocido. En la moto, tardaron una hora para llegar a [locality name redacted to protect confidentiality]. El curandero usó una hierba para saber que tipo de serpiente mordió a Gabriel, y cuando ya dijo que era una serpiente no tan venenosa, le receto Vaporub. Gabriel no recuerda cómo se llamaba la serpiente que le mordió, y perdió la foto.

“Yo soy de [locality name redacted to protect confidentiality] y vienen mis paisanos para trabajar aquí, y conozco gente que sí saben de serpientes. Conozco un hombre que venía, y él sabe curar al veneno. Me dijo que no era una serpiente venenosa y solamente me iba a dar calentura… Mi padre también sabía hacer eso, era tipo curandero como este hombre. Él sabe que hacer, es su trabajo. Busca hierbas, hay hierbas que conoce que son especialmente para serpientes.”

“Esta serpiente daba mucha calentura. Si era venenosa pero no mucho.”

“La gente tiene miedo de las serpientes. A veces los matan, a veces los corren.

“Primero fui a este señor [el curandero] para aguantar el viaje al hospital en [locality name redacted to protect confidentiality], porque el viaje es muy largo”
