# Supplementary material for: Therapeutic itineraries of snakebite victims and antivenom access in southern Mexico
Source: PLoS Negl Trop Dis. 2024 Jul 5;18(7):e0012301. doi: 10.1371/journal.pntd.0012301 (PMC11262687; doi:10.1371/journal.pntd.0012301)
Supplement: S1 Interview summaries — (ZIP) [file pntd.0012301.s002.zip › vasquez-neri-carter_2024_data_files/Interview Summaries/Interview Summaries/Javier.docx]

Javier, [locality name redacted to protect confidentiality], no se sabe cuando fue mordido, tenía 8 años

(Papa Carlos contando la historia)

Javier, un niño Tzotzil, fue mordido por un coralillo (uno de 5 especies de *micrurus* en Chiapas) cuando jugaba en la tierra, cavando un hoyo. Lo cubrieron con barro, porque creen que las serpientes coralinas no tienen cura. No tenía otros síntomas.

“El coralito ese no tiene remedio. Él no tomó nada. Lo embarramos de lodo.”

“Le pico la cola del coralito!”
