# Supplementary material for: Therapeutic itineraries of snakebite victims and antivenom access in southern Mexico
Source: PLoS Negl Trop Dis. 2024 Jul 5;18(7):e0012301. doi: 10.1371/journal.pntd.0012301 (PMC11262687; doi:10.1371/journal.pntd.0012301)
Supplement: S1 Interview summaries — (ZIP) [file pntd.0012301.s002.zip › vasquez-neri-carter_2024_data_files/Interview Summaries/Interview Summaries/Jorge.docx]

Jorge, [locality name redacted to protect confidentiality], mordido 2004, tenía 24 años

En 2004, Jorge, hombre de 24 años, estaba limpiando el camino en la granja de café, usando sandalias. Fue mordido en el dedo por una gran cola blanca, y 15 minutos después sintió que se le calentaba el dedo. No vio a la serpiente por la hierba. Se sentó por un minuto, pero no pudo levantarse. 15 o 20 minutos después ya no podía caminar. 30 minutos después de la mordedura, empezó a salir sangre de sus dientes. Llegó caminando desde la vereda y llegó a la clínica, donde le administraron un vial de antiveneno, por vía intramuscular.

“Esa pica con la cola. Me pico en el dedo. Dije ‘yo no voy a hacer nada, la neta’ porque no era grande. A los 15 minutos sentí que estaba caliente mi dedo. Lo sentía caliente, lo andaba acariciando y me senté. Pero a la hora que ya quería levantarme, ya no pude. Me puse muy garabato. Como unos 15, 20 minutos ahí estaba yo batallando y no podía caminar ya. A una media hora estaba saliendo sangre de mi dedo. Asi me vine caminando y me pase a la clinica aqui en [locality name redacted to protect confidentiality], y me pusieron una ampolleta. Porque hay ampolletas de medicina propensas para eso. Pero muchos ocupan café amargo, lo toman. A la gente que le gusta el trago también toman trago. Eso lo corta [al veneno] también. Ya me estaba afectando, y ahí estaba la clínica, entonces llegué. Cuando llegué a la clínica ya estaba yo grave. Me salió sangre de los dientes. Otro ratito más y creo que yo me iba a morir. Es que ese animal son 24 horas. Si no te ponen nada o tardas, se muere uno. Pero me pusieron la ampolleta y se me quito. A las 2 o 3 horas me sentí mejor. Cuando llegó la medicina, se cortó rápido. ”

“Algunos solo meten la mano en agua caliente y para su desgracia nada más se mueren.”
