# Supplementary material for: Therapeutic itineraries of snakebite victims and antivenom access in southern Mexico
Source: PLoS Negl Trop Dis. 2024 Jul 5;18(7):e0012301. doi: 10.1371/journal.pntd.0012301 (PMC11262687; doi:10.1371/journal.pntd.0012301)
Supplement: S1 Interview summaries — (ZIP) [file pntd.0012301.s002.zip › vasquez-neri-carter_2024_data_files/Interview Summaries/Interview Summaries/Jose.docx]

Jose, [locality name redacted to protect confidentiality], mordido 2021, tenía 9 años [interview with Jose's father]

Era el primer corte de café. José, un niño Tseltal de 9 años, estaba tirando hacia sí una rama de café y una víbora verde lo mordió en su mano derecha. Al principio pensó que era una abeja, pero la serpiente volvió a morderlo y vio que era una serpiente verde. Jose se fue para buscar a sus papás. Un amigo de la familia llevó a José en moto desde la granja a su casa [locality name redacted to protect confidentiality], donde el padre de José le hizo un torniquete en el brazo derecho. La familia pagó más por un taxi de emergencia, y condujo aproximadamente dos horas y media hasta el hospital de [locality name redacted to protect confidentiality]. Las enfermeras le administraron un “suero” y observaron si la hinchazón de José empeoraba o si ocurría alguna otra complicación. Le dieron de alta esa noche. El brazo de José permaneció hinchado durante 15 días.

(Padre de Jose contando) “En el momento que estaba jalando su rama, no se dio cuenta. Le mordió dos veces a la culebra. Se espantó, empezó a llorar. Nosotros (los papás) no vimos la culebra, pero el dice que era uno verde. Empezó a hinchar su brazo.”

“En este momento pagamos lo que es el taxi para 5 personas, tuvimos que pagar con tal de que uno llegue pues. Realmente no sabíamos que culebra era, entonces fuimos de emergencia.”

“No le pusimos nada, no sabemos, no hemos recibido de alguna platica de que se debe de hacer en caso de que le muerda a uno una serpiente.”

“Aquí según tenían antiveneno, pero cuando llegamos no había. A veces dan cada fin de mes, pero no lo tenían en la clínica.”

(Padre de Jose hablando) “Hay dos tipos de estos verdes, hay uno que es verde verde, y otro que tiene manchitas celestes. Dicen que este con manchas es más peligroso, a muchos les han picado. No muere uno, pero provoca mucho dolor. Pero Jose resistió, no sé si es mas fuerte.”
