# Supplementary material for: Therapeutic itineraries of snakebite victims and antivenom access in southern Mexico
Source: PLoS Negl Trop Dis. 2024 Jul 5;18(7):e0012301. doi: 10.1371/journal.pntd.0012301 (PMC11262687; doi:10.1371/journal.pntd.0012301)
Supplement: S1 Interview summaries — (ZIP) [file pntd.0012301.s002.zip › vasquez-neri-carter_2024_data_files/Interview Summaries/Interview Summaries/Juan Carlos.docx]

Juan Carlos, [locality name redacted to protect confidentiality], mordido 2021, tenía 58 años

Juan Carlos, trabajador migrante Guatemalteco de 48 años, estaba trabajando en el cafetal alrededor de las 9 a. m. del mes de julio de 2021 en la Finca [farm name redacted to protect confidentiality] cuando resbaló y se cayó. Puso su mano en el suelo para sostenerse, y fue mordido por una cola blanca (*Agkistrodon bilineatus* o *Bothrops asper*) en la palma de su mano. Juan Carlos le hizo un torniquete en el brazo para “detener la propagación del veneno”. 20 minutos después de la mordedura, se sintió mareado y le dolía la cabeza. Los dientes de Juan Carlos empezaron a sangrar. Su jefe en la plantación lo llevó, saliendo a las 10 de la mañana para ir al hospital de [locality name redacted to protect confidentiality], donde llegaron alrededor de las 11:30 am. Le inyectaron algo pero no sabe qué era. Alrededor de las 5 de la tarde, el hospital [locality name redacted to protect confidentiality] remitió a Juan Carlos al hospital de [locality name redacted to protect confidentiality] para recibir antídoto. A su llegada, el personal del hospital de [locality name redacted to protect confidentiality] no lo atendió, alegando que Juan no había sufrido una mordedura de serpiente venenosa. A Juan le sangraban los dientes y la nariz, pero la enfermera dijo que esto se debía a la mala higiene de Juan y que probablemente Juan no se cepillaba los dientes. Juan estaba molesto, regresó a [locality name redacted to protect confidentiality] y siguió sangrando por los ojos y los dientes hasta las 10 de la mañana del día siguiente, cuando lo soltaron para que se fuera a casa.

“Me mandaron para [otro hospital] en [locality name redacted to protect confidentiality] pero en [locality name redacted to protect confidentiality] no me atendieron porque dijeron que no era un animal peligroso. Yo dije que si era un animal peligroso, que me estaba sangrando la boca. Me dijo esa doctora que no, que yo estaba muy sucio y por esto estaba sangrando mi boca. Y no me creyeron… Me trasladaron otra vez para [locality name redacted to protect confidentiality], y empezó a sangrar más mi boca pero ya me habían dado de alta. Me enoje yo. No me atendieron ahí, y me dieron de alta en [locality name redacted to protect confidentiality]. Pero siguió sangrando mi boca.”

“Siente uno la muerte cuando le pica una culebra... Se sabe que este animal es venenoso. Se siente la muerte. Me espante, pensé, ‘Si no me sacan, me muero.’ Escupía bastante sangre, no poco, bastante.”

“Si me inyectaron allá (en el primer hospital) pero no se que me pusieron allá. Sí me sentí un poco mejor después, porque antes me sentía muy mareado.”

“Hay unos cantiles grandotes. Esos son más peligrosos todavía.”

“Un mes estuvo internado (otro compañero del trabajo). Sintió que (la serpiente) le dejó ir todo el líquido. Ahi si descargo lo que tenía.”

“Ahí hemos matado [a serpientes] y aparece uno ahí, y dice ‘ahí me lo llevo’ y dicen que lo miden. Toman un trocito de la cabeza y de la cola, y dicen que lo comen. Dicen que cura muchas enfermedades. hay otros que lo parten y ahí adentro hay un tipo de aceite. Le sacan todo el aceite. Lo meten en un frasquito y lo usan para curar la grasa. Una vez me lo quisieron dar pero no me dio el deseo de comerlo… La Santa Biblia dice que no está bueno comer la serpiente. Pero muchos lo comen para curar la enfermedad.”
