# Supplementary material for: Therapeutic itineraries of snakebite victims and antivenom access in southern Mexico
Source: PLoS Negl Trop Dis. 2024 Jul 5;18(7):e0012301. doi: 10.1371/journal.pntd.0012301 (PMC11262687; doi:10.1371/journal.pntd.0012301)
Supplement: S1 Interview summaries — (ZIP) [file pntd.0012301.s002.zip › vasquez-neri-carter_2024_data_files/Interview Summaries/Interview Summaries/Lorenzo.docx]

Lorenzo, [locality name redacted to protect confidentiality], mordido 2005, tenía 11 años

Alrededor de las 9 de la mañana en 2005, Lorenzo, un niño de 11 años estaba en la granja cuidando las plantas de maíz cuando una serpiente de cascabel, *Crotalus culminatus*, lo mordió en la parte interna del brazo. Se fue con su padre, 2 horas caminando hasta su casa en el [locality name redacted to protect confidentiality]. 1 hora y 40 minutos después de la mordedura, Lorenzo ya no podía caminar. Su padre lo cargó. Un vecino los llevó al centro de salud del municipio, pero le informaron a Lorenzo que no tenían antídoto. Su padre se comunicó con su amigo y escuchó que éste conocía a un médico en [locality name redacted to protect confidentiality] que tenía un frasco de antídoto. El padre de Lorenzo fue hasta allí y consiguió un vial de antiveneno por 580 pesos, para llevarlo al centro de salud del [locality name redacted to protect confidentiality] una hora después. El centro de salud inyectó a Lorenzo y lo retuvo durante la noche. Al día siguiente todavía le salía sangre de los ojos, los oídos y los dientes. Una enfermera quería abrirle el brazo para aliviar la presión de la inflamación, pero su padre no quería que esto sucediera. La víctima está feliz de no haber sido operado porque le habrían quedado cicatrices. Lo mantuvieron 6 días en la clínica porque todavía sangraba y a los 6 días lo enviaron a casa. Cuando se levantó para caminar 10 o 15 metros estaba cansado. Su papá fue y encontró otro antídoto para dárselo después de estos 6 días. Este antiveneno tuvo menos efecto y el padre finalmente encontró un tercero. Por cada antídoto pagaron 580 pesos.

“Llegamos rápido a la casa, y pedimos el favor de un vecino que nos llevará al centro de salud en su moto. Pero al llegar al centro de salud, nos informaron que no contaban con el antídoto. Entonces mi padre lo que hizo fue comunicarse con su compadre. Y el amigo le dijo que conocía a un doctor en Pijijiapan que tenía un antídoto, entonces el compadre pidió el antídoto, pero en ese entonces yo ya tenía el brazo inflamado. Prácticamente el veneno ya… casi me desmayaba.”

“Después de que me inyectaron el antídoto, me costó recuperarme. Estuve 6 días en observación porque mis ojos me salieron sangre. Me sangraron, y las sencillas, los oídos. Ya estaba muy avanzado el veneno. Lo que hizo mi padre fue trasladarme a casa y estuve en reposo mínimo un mes. Caminaba unos 5 metros y sentía que el corazón todavía no me funcionaba correctamente. Entonces todavía me aplicaron otro pero ya era tarde. Por fin me aplicaron como 3, y todos costaban como 580 pesos.”

“Había una enfermera aquí que por ignorancia, o no se, que bueno que mi padre no accedió, quería zanjar el brazo. Me quería sacar todo lo que era la inflamación. La quería zanjar y extraer todo eso. Si eso hubiera pasado ya tendría grandes cicatrices. Sería más complicado.”

“Para comprar el antídoto, no es nada fácil. Es muy difícil conseguirlo. No hay.”
