# Supplementary material for: Therapeutic itineraries of snakebite victims and antivenom access in southern Mexico
Source: PLoS Negl Trop Dis. 2024 Jul 5;18(7):e0012301. doi: 10.1371/journal.pntd.0012301 (PMC11262687; doi:10.1371/journal.pntd.0012301)
Supplement: S1 Interview summaries — (ZIP) [file pntd.0012301.s002.zip › vasquez-neri-carter_2024_data_files/Interview Summaries/Interview Summaries/Luis.docx]

Luis, [locality name redacted to protect confidentiality], mordido 1978, 1987, y 1993, cuando tenía 10, 19 y 25 años

Luis fue mordido 3 veces por Bothriechis bicolor, “cotorrera”. La primera vez, tenía 10 años en 1978, cosechando café en [locality name redacted to protect confidentiality], cerca de [locality name redacted to protect confidentiality], cuando sintió la picadura en su mano. Agitó el brazo para quitarse la serpiente y su jefe mató a la cotorrera. Luis se asustó y se sentó a descansar. Después de 2 horas, Luis todavía no sentía síntomas de intoxicación, por lo que comenzó a trabajar nuevamente. Al final del día, alguien preguntó: “¿A quién mordió la serpiente?” y Luis dijo: “¡Yo!” y le dieron un poco de brandy.

“La primera vez que tenía 10 años, y todas las serpientes eran venenosas para mi. Cualquiera iba a matar. Pero después del piquetazo, a las 2 horas no me hizo nada. Empecé a cortar café todavía. En la tarde dijeron, ‘¿a quien le picó la culebra?’ Y dije ‘yo’ y me dieron un poco de trago. Pero ya había pasado.”

La segunda mordida ocurrió cuando Luis tenía 19 años en 1987, cortando palma camedor en [locality name redacted to protect confidentiality]. Fue mordido en la misma mano, también por un Bothriechis bicolor. Dice que no pasó nada.

La tercera mordida ocurrió cuando Luis tenía 25 años en 1993, cortando palma camedor en [locality name redacted to protect confidentiality]. Una vez más, no sintió síntomas de envenenamiento. Luis comenzó a decirle a la gente que las serpientes verdes no son venenosas y que la gente no debe preocuparse si las muerden.

Una vez, alguien de la finca fue mordido por la serpiente y Luis dijo que no se preocupen. El trabajador tenía dolores, entonces Luis le encontró alcohol y el otro trabajador se emborrachó. El brazo del trabajador se hinchó. Luis dice que tal vez sea porque la serpiente no había comido.

“La primera vez sentía miedo, y la segunda vez no me dio miedo porque sé que no pasa nada. La tercera menos. Hay otra persona que estaba cortando palma ahí en el [locality name redacted to protect confidentiality], y le pico la culebra. Me dijo ‘me voy a morir’ y le dije ‘no le pasa nada, esa serpiente no es tan venenosa.’ ‘Pero me duele’ me dijo él. Entonces le di un litro de trago y se puso bien borracho. Se le hincho bien, se le hincho la mano. Estaba sangrando… No se porque, tal vez no había comido la serpiente, no se.”

“Hay otros que sí le da cosa. Hay otro amigo que se le mordió, y no le hice caso porque ya me había mordido. Pero se le hincho, estaba de reposo unos 5, 6 días… Hay personas con sangre más fuerte, como yo que no me afecta la serpiente.”
