# Supplementary material for: Therapeutic itineraries of snakebite victims and antivenom access in southern Mexico
Source: PLoS Negl Trop Dis. 2024 Jul 5;18(7):e0012301. doi: 10.1371/journal.pntd.0012301 (PMC11262687; doi:10.1371/journal.pntd.0012301)
Supplement: S1 Interview summaries — (ZIP) [file pntd.0012301.s002.zip › vasquez-neri-carter_2024_data_files/Interview Summaries/Interview Summaries/Manuel.docx]

Manuel, [locality name redacted to protect confidentiality], mordido 2020, tenía 34 años

En Abril de 2020, Manuel, un hombre Tzotzil de 34 años, estaba agarrando agua para la finca cafetalera. Puso su mano sobre una piedra, cuando fue mordido en la muñeca por una cotorrera, *Bothriechis bicolor*. Vio las marcas de los colmillos y la sangre. No usó ningún medicamento y siguió trabajando.

“No sentí dolor, no me puse nada. Depende la reacción de cada cual, supongo.”
