# Supplementary material for: Therapeutic itineraries of snakebite victims and antivenom access in southern Mexico
Source: PLoS Negl Trop Dis. 2024 Jul 5;18(7):e0012301. doi: 10.1371/journal.pntd.0012301 (PMC11262687; doi:10.1371/journal.pntd.0012301)
Supplement: S1 Interview summaries — (ZIP) [file pntd.0012301.s002.zip › vasquez-neri-carter_2024_data_files/Interview Summaries/Interview Summaries/Marco.docx]

Marco, [locality name redacted to protect confidentiality], mordido 2015, tenía 22

Marco (hijo de Antonio) fue mordido en 2015 en la rodilla por una sorda, alrededor de las 7 de la tarde, cuando limpiaba el pasto alrededor de una cerca en la finca de hule. El jefe de Marco lo llevó al hospital de [locality name redacted to protect confidentiality], donde pasó la noche. Marco estuvo vomitando sangre durante la noche. El hospital de [locality name redacted to protect confidentiality] no tenía antídoto, por lo que lo derivaron a [locality name redacted to protect confidentiality] alrededor de las 7 de la mañana. Llegaron aproximadamente una hora después en una ambulancia pagada por el hospital y Marco recibió el antiveneno. Permaneció 3 meses en el hospital de [locality name redacted to protect confidentiality] como paciente internado. Durante los primeros 15 a 20 días, Marco no podía orinar y sangraba por los ojos, los dientes, la nariz y los oídos. Marco ahora es estéril. Marco sólo fue al hospital porque don pepe, el curandero local, estaba muerto. Dicen que Don Pepe cura más rápido porque le quita el veneno, mientras que en el hospital sólo le inyectan medicina.

“En esos tiempos era canícula. Todo lo que te pique te sale pus. Lo que se corte lo va a punzar. Por eso el calor está así ahora. Cómo es tiempo de calor pero el calor es por la canícula… Cuando llegamos al hospital, si hubo la dosis pero no hubo completo. No era suficiente. Ya no lo consiguieron entonces lo trasladaron a [locality name redacted to protect confidentiality]. Y en [locality name redacted to protect confidentiality] si había. Lo pico a las 6 de la tarde y fuimos a [locality name redacted to protect confidentiality]. Fuimos a las 6 de la mañana de [locality name redacted to protect confidentiality] en ambulancia. De un solo piquete estuvo como 6 meses ahí internado, porque estuvo unos 15 o 20 días sin poder orinar. Y sacó sangre por todos los poros, por los ojos, la nariz, las orejas, las encías. Eso lo espanto. Eso fue cuando Don Pepe ya no estaba. Su hijo también curaba pero murió en la pandemia.”
