# Supplementary material for: Therapeutic itineraries of snakebite victims and antivenom access in southern Mexico
Source: PLoS Negl Trop Dis. 2024 Jul 5;18(7):e0012301. doi: 10.1371/journal.pntd.0012301 (PMC11262687; doi:10.1371/journal.pntd.0012301)
Supplement: S1 Interview summaries — (ZIP) [file pntd.0012301.s002.zip › vasquez-neri-carter_2024_data_files/Interview Summaries/Interview Summaries/Mario.docx]

Mario, [locality name redacted to protect confidentiality], mordido 2016, tenía 41 años

Mario tenía 41 años el 25 de agosto de 2016 cuando estaba cazando jabalíes al otro lado de un río cerca a [locality name redacted to protect confidentiality]. De regreso, Mario estaba con sus amigos, y todos estaban borrachos. Mario ya se había tomado un litro, y tenía otro en su mochila. Mario sintió la mordedura en su pierna y vio la serpiente de cascabel, Crotalus culminatus. Dice que tenía alrededor de 13 agitadores en el sonajero pero que no había hecho ningún ruido cuando venían. Mario llevaba botas y pantalones, por lo que sólo un colmillo se deslizó por su bota. Inmediatamente bebió la media botella de aguardiente que le quedaba y caminó hacia el pueblo, [locality name redacted to protect confidentiality]. Después de aproximadamente una hora de caminata, había una costra de sangre sobre la marca del colmillo. Rompió la costra y lavó la herida con gasolina, antes de continuar su caminata. Desde que Mario fue mordido hasta su llegada a [locality name redacted to protect confidentiality] pasaron 1.5 horas. Estaba borracho y fue a buscar más aguardiente. Unos vecinos le dieron media botella de tequila y él se la bebió. También tomó un litro de aguardiente y se lo bebió. Dice que llegó borracho al hospital de [locality name redacted to protect confidentiality] y que los médicos no tuvieron que hacer mucho porque el alcohol lo había salvado. Le dieron paracetamol para el dolor y dicloxacilina para prevenir cualquier infección. Dice que le dieron “puro suero”, probablemente refiriéndose a una solución salina. Mario advirtió que, según las creencias locales, tanto el veneno como el suero son fríos, por lo que el suero no ayuda a curar el veneno. El alcohol se considera “picante”, por lo que cree que ayuda más. El hospital hizo 5 o 6 análisis pero no encontraron veneno porque el alcohol lo había neutralizado, según Mario. Toda su pierna estuvo hinchada durante unos 20 días y se infectó. Después de 10 días, algunos amigos le dijeron que bebiera “hormiguillo”, una hierba, con aguardiente. Mario afirma que eso ayudó a reducir el dolor y la inflamación. Dijo que tenía un contacto en otro hospital, una enfermera que le ofreció venderle un antídoto por 8000 pesos si lo necesitaba.

Mario cuenta que hace 12 años, un amigo llamado Federico (que entonces tenía 32 años) iba caminando a Palestina y lo mordieron en el camino. Una vez que Federico llegó a [locality name redacted to protect confidentiality], llegó al hospital y se desmayó. Le sangraron los dientes durante casi 2 años cuando comía cosas frías.

“Me di cuenta por el dolor. Solo una comilla entró porque tenía botas. Duele el veneno. Me trage medio botella de aguardiente, y con esto llegué aquí a la colonia [locality name redacted to protect confidentiality], pero mareado, nada. Fui a un amigo que vendía alcohol, y me dio un cristal de tequila y me lo eché también. Mi compañero tenía otro litro de caña, y lo heche también. Eran tres tragos. Cuando llegué al hospital estaba más borracho por el alcohol. Lo que me espanto fue el doctor que me mando 3 inyecciones contra el veneno, porque si al otro día había veneno, me iban a cortar mi rodilla. Pero una enfermera me dijo que si exigían, ella tenía una vacuna [un antiveneno], costaba 8 mil pesos, pero no hubo necesidad. Con el puro alcohol se cortó el veneno. Suero me pusieron, pero por experiencia.. no quería que me pusieran el suero porque el suero es frío y el veneno de la culebra es frío también. Así que ayuda mas el trago al veneno porque el trago es caliente. Y si el alcohol corta el veneno, porque me hicieron 5 análisis, 3 de pipi y 2 de sangre y nunca hubo veneno de víbora.”

“camine como una hora [después del accidente] y llegue a donde trabajaba yo, y tenía gasolina. Y ahí tenía como una bomba de sangre seca. Y lo rompí con espina, lo lave con gasolina y con esto aguante llegar. No tenía más alcohol.”

“Un compadre perdió el camino hacia [locality name redacted to protect confidentiality], y le mordió la víbora. Se desmayó él cuando llegó al hospital, y le pusieron suero. Dicen que tardó como dos años que le sangraron las sencillas cuando tomaba refresco frío o cuando comía un helado, salía sangre. El si lo sufrió. Dice que es porque pasó el agua del río.”

“Se me hincho hasta la cintura. Y bajó la hinchazón como a los 20 días. A las 10 días me dijeron que tome agua de hormiguillo, de la cáscara de hormiguillo con trago. Y eso ayudó a espantar el dolor porque me dolía.”
