# Supplementary material for: Therapeutic itineraries of snakebite victims and antivenom access in southern Mexico
Source: PLoS Negl Trop Dis. 2024 Jul 5;18(7):e0012301. doi: 10.1371/journal.pntd.0012301 (PMC11262687; doi:10.1371/journal.pntd.0012301)
Supplement: S1 Interview summaries — (ZIP) [file pntd.0012301.s002.zip › vasquez-neri-carter_2024_data_files/Interview Summaries/Interview Summaries/Martin.docx]

Martin, [locality name redacted to protect confidentiality], mordido 2022, no se sabe cuántos años tenía

Martin, estaba cuidando los cafetos en mayo 2022 cuando le mordió un cantil en la muñeca, intentó matar a la serpiente pero no pudo. El brazo sangraba mientras caminaba 2 horas de regreso a [locality name redacted to protect confidentiality]. Casi se desmaya durante la caminata, pero mantuvo la conciencia. Se ató un torniquete alrededor del brazo para evitar que el veneno se esparciera. Fue al hospital de [locality name redacted to protect confidentiality] (a 1,5 horas de [locality name redacted to protect confidentiality]) en su motocicleta, donde le dieron uno o dos viales de antiveneno (no está seguro porque no estaba del todo consciente). De allí lo derivaron a [locality name redacted to protect confidentiality] (4 horas en ambulancia desde [locality name redacted to protect confidentiality]). Ahora sufre daños en los tendones y no puede doblar los dedos. 4 días en el hospital, Después, siguió una dieta sin grasas y sin huevos.

(Habla el vecino) cuando a uno de nosotros nos muerden, tenemos que unirnos todos y ayudar a conseguirle un remedio casero para que sobreviva al veneno.

“Estaba yo trabajando, sembrando café. Me descuide. Cuando sentí la mordedura, como espina se ve. Me quedé mirando mi mano. Estaba saliendo sangre. Me andaba tirando ahí, por poquito no me desmayé arriba. Apenas aguanté a venir aquí en la colonia. Son dos horas caminando, y me vine rápido, me venía tembloroso. dije, ‘yo voy a morir aquí’. Pero fue una bendición de dios que no… pero no quedó bien mi mano. ¡No puedo agarrar nada! Me hormiguea, como zanahoria lo siento adentro. Feo lo siento, no es normal.”

“Cuando me mordió, me apreté con un pañuelo para que no corriera el veneno. Sino, me hubiera corrido el veneno. No tomé nada, nomas me inyectaron cuando llegué a [locality name redacted to protect confidentiality]. Nomas me pusieron uno, o a lo mejor dos ampolletas. No me acuerdo yo, no me di cuenta que estaba yo como que muerto.”

“Me sacaron varias jeringas de sangre, y yo me daba lastima para mi sangre.”
