# Supplementary material for: Therapeutic itineraries of snakebite victims and antivenom access in southern Mexico
Source: PLoS Negl Trop Dis. 2024 Jul 5;18(7):e0012301. doi: 10.1371/journal.pntd.0012301 (PMC11262687; doi:10.1371/journal.pntd.0012301)
Supplement: S1 Interview summaries — (ZIP) [file pntd.0012301.s002.zip › vasquez-neri-carter_2024_data_files/Interview Summaries/Interview Summaries/Mateo.docx]

Mateo, [locality name redacted to protect confidentiality], mordido 1977, tenía 12 anos

Mateo tenía 12 años cuando fue mordido por una Nauyaca de Frío, *Cerrophion godmani* en 1977*.* En esta temporada pasaban 800-900 personas indígenas haciendo un peregrinaje anual hacia San Cristóbal de las Casas. Uno de estos peregrinos pidió posada en la casa de Mateo. Su familia de Mateo había salido a campo para el Pacífico para dejar pastar a su ganado, pero Mateo se quedó en casa para cazar pájaros. Mateo y su compañero estaban en el bosque cuando vieron un pájaro que se aterrizó en un árbol caído, y Mateo lo fue a buscar. Metió su mano para buscar en el árbol, y una serpiente le mordió en su mano. Dice que se sintió como una picadura de araña. A las 9 de la mañana fue mordido, y fue corriendo a su casa, donde se estaba quedando el peregrino. Llegó a las 10 de la mañana y le contó al peregrino que le había mordido una serpiente. Mateo sintió mareo y su brazo estaba hinchado. “[el Don me dio cedrón… es una vejuca que es buena como la viborina. La viborina es buena.”

El hombre indigena le amarró dos torniquetes a Mateo, y succiono la herida con tabaco en su boca. El peregrino le cortó la herida con una navaja, y sangro mucho. Mateo pensó que se iba a morir.

“Este caminante me salvó, me mejoró el dolor… La gente del campo tiene bastante conocimiento.”

“No pasaba el dolor, el mundo daba vueltas. Me sentía muy mal, y doy gracias a este señor”

(pregunta: Había hospital cerca?)

Mateo: “No, no no no. En estos días no había nada. Mi brazo estaba hinchado por un mes y medio porque no me estaba inyectando nada. No había ni una pastilla. Usaba más plantitas. Sí había medicina pero costaba caro. Tardó bastante en sanar.”

En los días después del accidente, Mateo frotó posos de café en la herida y siguió una dieta especial que le dio el anciano, que prohibía la carne, la leche, y (en el momento después de la mordida) agua. Su dieta después del accidente estaba compuesta principalmente de café negro y tostadas. Su brazo estaba hinchado por un mes y medio.

“Casi confio mas en plantas que los médicos, porque la medicina te cura una parte y el otro parte te hace mal”
