# Supplementary material for: Therapeutic itineraries of snakebite victims and antivenom access in southern Mexico
Source: PLoS Negl Trop Dis. 2024 Jul 5;18(7):e0012301. doi: 10.1371/journal.pntd.0012301 (PMC11262687; doi:10.1371/journal.pntd.0012301)
Supplement: S1 Interview summaries — (ZIP) [file pntd.0012301.s002.zip › vasquez-neri-carter_2024_data_files/Interview Summaries/Interview Summaries/Matias.docx]

Matias, [locality name redacted to protect confidentiality], mordido 2017, tenía 77 años

Matías caminaba de regreso del trabajo en una finca cafetera. Una gran serpiente de cascabel, *Crotalus culminatus*, estaba al costado del camino, pero Matías no la vio debido al follaje. La serpiente le mordió la rodilla. Caminó desde el lugar donde fue mordido hasta su casa, unos 1500 metros. Tuvo que pasar el río en la caminata, y Matías dice que pasar el agua empeoró sus síntomas porque lo golpeó el frío (y el veneno de serpiente se considera resfriado en la medicina tradicional). En casa de Matías tomó aguardiente y puso torniquete. Luego sus vecinos lo llevaron al hospital de [locality name redacted to protect confidentiality], donde recibió 9 viales de antídoto. Los médicos querían darle uno más pero no tenían suficiente. Matías salió del hospital al día siguiente. Todavía siente dolor en la rodilla.

“Me dijo el médico que me atendió que por suerte viví, porque me pegó el frío del agua del río. Pero aquí estamos todavía.”

“Me picaron 9 frascos de medicina. Me dijeron que me faltaba otro pero ya no tenían.”
