# Supplementary material for: Therapeutic itineraries of snakebite victims and antivenom access in southern Mexico
Source: PLoS Negl Trop Dis. 2024 Jul 5;18(7):e0012301. doi: 10.1371/journal.pntd.0012301 (PMC11262687; doi:10.1371/journal.pntd.0012301)
Supplement: S1 Interview summaries — (ZIP) [file pntd.0012301.s002.zip › vasquez-neri-carter_2024_data_files/Interview Summaries/Interview Summaries/Miguel Angel.docx]

Miguel Angel, [locality name redacted to protect confidentiality], mordido 2008, tenía 59

Miguel Angel estaba limpiando plantas de café con una machete y un cantil, *Agkistrodon bilineatus*, le mordió en su mano derecha. Se fue a la clínica del [locality name redacted to protect confidentiality] pero no había nadie. El hermano fue a buscar el antiveneno. El hermano de Miguel Angel encontró un frasco de antiveneno 2 horas después, en un rancho familiar cerca de [locality name redacted to protect confidentiality]. El hermano lo trajo a casa. No había personal médico en el pueblo, por lo que un vecino le inyectó el antídoto a Alberico. Siguió una dieta especial durante 40 días, sin carne, sin aceites ni grasas.

“Me inyectaron aquí. Una persona me inyectó particularmente. No había la unidad médica.”

“Son 40 días de dieta. No se puede comer carne, nada de grasa…”
