# Supplementary material for: Therapeutic itineraries of snakebite victims and antivenom access in southern Mexico
Source: PLoS Negl Trop Dis. 2024 Jul 5;18(7):e0012301. doi: 10.1371/journal.pntd.0012301 (PMC11262687; doi:10.1371/journal.pntd.0012301)
Supplement: S1 Interview summaries — (ZIP) [file pntd.0012301.s002.zip › vasquez-neri-carter_2024_data_files/Interview Summaries/Interview Summaries/Miguel.docx]

Miguel, [locality name redacted to protect confidentiality], mordido 2002, tenía 17 años

Miguel, hombre Tzotzil del ejido [locality name redacted to protect confidentiality], tenía 17 años cuando fue mordido en el año 2002. Estaba caminando hacia su trabajo, alrededor de las 7:30 am cuando un *Bothriechis bicolor*, Cotorrera lo mordió en el brazo. En casa, el padre de Miguel cortó la herida con una espina, creyendo que el veneno se escaparía con la sangre. Unos miembros de la familia mezclaban creolina con hojas de sábila verde (lengua de suegra) y él la bebía. La familia le hizo un torniquete a Miguel y este bebió aguardiente. Dos horas más tarde, sintió un gran dolor. Sintió como si el veneno y el dolor se dirigieran hacia su corazón. Comió un plato de chile seco y bebió agua hirviendo. Al día siguiente tenía todo el brazo hinchado y así permaneció durante 7 días, después de lo cual empezó a trabajar para poder sudar el resto del veneno. Durante los primeros días de trabajo todavía le dolía la mano.

“Aquí los hemos tratado con hierbas y alcohol, hasta posh [un alcohol fuerte de maíz], porque son animales venenosos.”

“Tratamos de sacar el veneno pero no se pudo. Vine para mi casa, llega mi papá, y me rajaba un poco con una espina, pero no pudieron sacar nada. Al momento que me pico, lo amarramos con un pañuelo. Llegaron mis familiares y empezaron a dar una hoja que es creolina. Lo molieron con un tipo sabila, y me lo dieron con aguardiente. Empecé a tomar porque dicen que es muy bueno, hay que controlar [el veneno]. También tomé un plato de chile seco y agua calientita. Después de dos horas, empezó un dolor bien fuerte que el veneno fue buscando mi corazón. Se siente bien feo. Al siguiente día se me hincho, se puso hasta el codo, la mano bien hinchada. Hice ejercicio para que sudara. A los 6 días me controlo, pero todavía me dolía mi mano y no podía trabajar unos días más.”

“Un viajecito [a la clínica o hospital] ya es un gasto. Un carro de aquí a [locality name redacted to protect confidentiality] son 600 pesos. A [locality name redacted to protect confidentiality] son 1,500 pesos, 1,200 pesos, depende. Ahora tengo una moto para moverme, pero hay personas que no tienen esa facilidad o no cuentan con este recurso más en este tiempo. Entonces, se quedan aquí buscando la solución que es más buena.”
