# Supplementary material for: Therapeutic itineraries of snakebite victims and antivenom access in southern Mexico
Source: PLoS Negl Trop Dis. 2024 Jul 5;18(7):e0012301. doi: 10.1371/journal.pntd.0012301 (PMC11262687; doi:10.1371/journal.pntd.0012301)
Supplement: S1 Interview summaries — (ZIP) [file pntd.0012301.s002.zip › vasquez-neri-carter_2024_data_files/Interview Summaries/Interview Summaries/Natalia.docx]

Natalia, [locality name redacted to protect confidentiality], mordida 2008, tenía 38 años

(esposo Carlos contando la historia) Natalia, mujer Tzotzil de 38 años, fue mordida en mayo de 2008, alrededor de las 11 de la mañana. Estaba desyerbando el cafetal cuando un “tamagaz”, *Cerrophion godmani*, la mordió en el tobillo. Natalia tomó un vaso de puré de chile en la montaña de la finca, luego ella y su esposo llegaron caminando a la casa. En la casa bebieron aguardiente. “No sé si había cura para la mordedura de serpiente en ese momento”, dijo Carlos.

“Normalmente bebemos chile licuado, así que eso fue lo que ella hizo.”

“Somos campesinos, no sabemos con qué curar las mordeduras de serpiente”.
