# Supplementary material for: Therapeutic itineraries of snakebite victims and antivenom access in southern Mexico
Source: PLoS Negl Trop Dis. 2024 Jul 5;18(7):e0012301. doi: 10.1371/journal.pntd.0012301 (PMC11262687; doi:10.1371/journal.pntd.0012301)
Supplement: S1 Interview summaries — (ZIP) [file pntd.0012301.s002.zip › vasquez-neri-carter_2024_data_files/Interview Summaries/Interview Summaries/Omar.docx]

Omar, [locality name redacted to protect confidentiality], mordido 2020, tenía 35 años

(entrevista con padre) Omar fue mordido en 2020, tenía 35 años y estaba instalando un poste en la finca cafetalera cuando pisó la serpiente cascabel, *Crotalus culminatus* y le mordió la pantorrilla izquierda. El jefe de la finca llevó a Omar al hospital de [locality name redacted to protect confidentiality] en su auto, pero no había antiveneno. Entonces, Omar fue remitido al hospital de [locality name redacted to protect confidentiality], a 1,5 horas en auto de [locality name redacted to protect confidentiality]. No había ambulancia, así que fueron en su coche. Omar recibió antiveneno a su llegada. 6 días en el hospital de [locality name redacted to protect confidentiality].

“Se le mordió bien feo. Es peligrosa una mordedura”

“Lo pusieron medicina pero no era la mera buena”
