# Supplementary material for: Therapeutic itineraries of snakebite victims and antivenom access in southern Mexico
Source: PLoS Negl Trop Dis. 2024 Jul 5;18(7):e0012301. doi: 10.1371/journal.pntd.0012301 (PMC11262687; doi:10.1371/journal.pntd.0012301)
Supplement: S1 Interview summaries — (ZIP) [file pntd.0012301.s002.zip › vasquez-neri-carter_2024_data_files/Interview Summaries/Interview Summaries/Osmar.docx]

Osmar, [locality name redacted to protect confidentiality], mordido 2003, tenía 47 años

**Osmar es diabetico

Osmar estaba cazando jabalíes en el campo de maíz alrededor de las 2 o 3 de la tarde con dos amigos. El maíz estaba cortado y recién comenzaba a crecer nuevamente. Osmar estaba descalzo cuando una Sorda hembra lo mordió en el dedo gordo del pie (dice que la serpiente era amarilla y algunos la llaman Palanca, señaló Porthidium dunni, pero esta serpiente no está presente en la region). Sus dos amigos le rasgaron la camisa y le hicieron dos torniquetes en el muslo y la rodilla. Un amigo bajó la carne de jabalí, mientras el otro acompañaba a Osmar. Mientras caminaban hacia el pueblo, se sintió mareado. A Osmar le sangraban los ojos y los dientes y orinó sangre. Cuando llegaron a [locality name redacted to protect confidentiality], Osmar se desmayó. Su amigo encontró una plataforma rodante para llevar a Osmar a la carretera, donde paró un taxi. El amigo llevó a Osmar en el taxi (30 pesos) a Don Pepe, un curandero local. Su pie empezó a ponerse negro y Don Pepe le dijo que no se preocupara, que eso era normal. El curandero cortó entre las dos marcas de los colmillos con un colmillo de serpiente, de modo que la herida sangraba. Entonces Don Pepe succionó el veneno. Don Pepe le dio una bebida amarga de hierbas. Don Pepe preguntó por la esposa de Osmar, quien en ese momento tenía 5 meses de embarazo. Don Pepe le dijo a Osmar que se asegurara de que su esposa no viniera a visitarlo, porque las víctimas de mordeduras de serpiente que ven a una mujer embarazada no pueden sanar. Estuvo poco más de 3 meses con Don Pepe. Para evitar ver a su esposa embarazada, Osmar se quedó con un amigo de la familia por otros dos meses, donde Don Pepe lo visitaba diariamente. Algunos amigos vinieron a visitarlo, incluida una mujer embarazada de tres meses en la casa de Don Pepe, pero él no se dio cuenta de que la visitante estaba embarazada hasta que ella se lo dijo más tarde. A 5 meses de la mordedura, Don Pepe dijo que no podía hacer nada más por Osmar. Don Pepe cobró 5500, sin incluir el transporte y la comida de Don Pepe. Existe la creencia local de que los curanderos deben comer antes de atender a un paciente. La herida aún no estaba curada. Sus pies estaban infectados y tenían úlceras. Fui a ver a un médico en [locality name redacted to protect confidentiality], para que le atendiera el pie. Le daba nervios mostrar el pie hasta saber cuánto le cobraría el médico, porque Osmar no tenía mucho dinero. El médico le aseguró a Osmar que no le cobraría mucho. El médico dijo que el pie estaba bien porque no había mal olor, pero la herida simplemente no había sanado. El médico le recetó un ungüento y le inyectó a Osmar un antibiótico para detener las infecciones. Osmar no podía caminar debido al dolor, por lo que el médico le recomendó reposo en cama hasta que su pie sanara. Pasaron otros 20 días después de visitar al médico y la herida todavía tenía pus. Otro amigo lo visitó y le preguntó si Osmar había visto a una mujer embarazada. Osmar le informó al amigo que una mujer embarazada había venido a visitarlo. El amigo dijo que para curar la herida, Osmar necesitaba que una mujer embarazada escupiera en su herida por mordedura de serpiente. Osmar llamó a su hermana embarazada para que viniera y le pusiera saliva en la herida con los dedos, y tres días después su herida mejoró.

“Me mordió la serpiente, y se puso negro mi piel. Pero me dijo el culebrero que no me espantara porque era normal que pase esto, y que me iba a sacar el veneno. El [culebrero] tiene los colmillos de la culebra, y lo corta donde hay la herida. Y sangra, y cuando sangra, el jala con la boca para sacar el veneno.”

“dije a mis compañeros, ‘Rompen mi playera’ y lo hicieron. Y uno me amarro aquí, y el otro me amarro aquí”

“Ya me sentía que estaba borracho. En mero cerro me estaba mareando. Llegando a Acatlán, perdí el conocimiento. Me llevaron en un burrito, y pasaba el taxi, y me llevaba con el culebrero.”

“Si tu vas al doctor, no te va a atender en seguida. Así es con Don Pepe. Si llega otro antes que tú, tienes que ponerte en cola. Y yo desesperado, dije ‘nombre, fue mordido por culebra, el veneno… no me voy a aguantar. Me voy a morir pues’. Como una hora espere, y me dio ‘toma de hierba’. Para que el veneno no siga corriendo en el cuerpo.”

“El defecto fue que me dijo, ‘¿cómo se mira tu esposa?’ Y yo le dije, ‘Mi esposa está gorda. Está cargando un bebe.’ Y me dijo, ‘No vaya a venir tu esposa. Por favor, mandala razón que no venga. Si esta gorda, te puede afectar el pie o puede fallecer.’ Entonces un muchacho la dijo. Vinieron hombres, vinieron mujeres para verme. Pero una de las mujeres me fue a ver y esta muchacha estaba gorda… 3 meses y medio estaba en la casa de Don Pepe, y no me podía curar. No me podía sanar la herida.”

“El puro dinero que me cobró de curar fueron 5,500, no contando pasaje y comida. Me daba de comer. Mi pie estaba llorando, y me dijo mi amigo, ‘para comprobar que tu pie no está llagoso, mejor vete con el médico.’. Yo fui, y dije al doctor, le dije ‘antes que todo, yo respeto su trabajo pero yo no traigo suficiente dinero. Mi problema es que me mordió una culebra, y no se cuanto vaya a salir que usted checa mi pie y me dice si me va a recuperar el pie o ya no.’ Dice el médico ‘No te preocupes, si no te alcance, no hay ningún detalle. Lo importante es que tú quedes bien.’ Entonces ya lo vio y me dijo que todo estaba bien. Me inyecto, por si alguna cosa, para que no me caiga la infección. No pude caminar casi, le dije ‘cuando bajo mi pie, se me pone morado. Y tiene mucho tiempo así.’ Y me dijo, ‘cuando bajas tu pie la sangre empieza a circular. No camines mejor. Solamente vas a levantarte cuando vas a hacer tus necesidades. Pon tu pie para arriba.’ Estaba en la hamaca, y parece que ya [se iba a sanar], no se secaba por otro 20 días. Uno me dijo ‘De casualidad no te vio una mujer gorda?’ Y le dije, ‘Ya que lo dices, la hermana de Saul ya tiene 5 meses que me fue a ver, y ya tiene 5 meses’ ‘Eso es la clave’ me dice, ‘que te echa un poco de su saliva en ese punto donde no cierra la herida. Ve a ella que te haga este trabajito’ Y ya me fui, y me hecho saliva tantito en mi herida, y a las 3 dias esta seco.”

“Primero fui a ver Don Pepe, y el costo de su trabajo fue 5,500, y aparte su pasaje y su comida. Después fui a los 5 meses al doctor, Y al último la que me cerró la herida fue la mujer gorda. Si me hizo el doctor, me dio la inyección y todo eso, pero en total eran 3 personas; el culebrero, el doctor, y la señora gorda.”

“Cuando le muerde una culebra a una persona, no puede ver a una señora gorda. Eso es lo que recomienda el culebrero.”

“Orinaba sangre, escopia sangre, perdí la vista, todo sangre.”
