# Supplementary material for: Therapeutic itineraries of snakebite victims and antivenom access in southern Mexico
Source: PLoS Negl Trop Dis. 2024 Jul 5;18(7):e0012301. doi: 10.1371/journal.pntd.0012301 (PMC11262687; doi:10.1371/journal.pntd.0012301)
Supplement: S1 Interview summaries — (ZIP) [file pntd.0012301.s002.zip › vasquez-neri-carter_2024_data_files/Interview Summaries/Interview Summaries/Perros- 1 fallecido, 1 tratado con antiveneno.docx]

[locality name redacted to protect confidentiality] - (Padre de Adrian) “Una noche, el perro estaba ladre y ladre.. llegue y la serpiente lo agarró en la oreja. Y gritó [el perro] y me avente. 1m70 media, así era el cascabel. El perro se fue, se perdió, se desubicó de su lugar. Lo mate a la serpiente y me lo lleve para la casa. Fui de regreso, para buscar el perro y lo vi botado, cerca de la casa. Rápido fue, dentro de 20 minutos. Hay muchas [serpientes] aquí en el rancho.”

[locality name redacted to protect confidentiality] - (Jefe de Enrique) “También teníamos un perro. Estaba aquí y le mordió una culebra y dio un grito. Lo llevamos y le dimos una inyección y por eso se salvó. Se salvó el perrito. Aquí hay muchas serpientes.”
