# Supplementary material for: Therapeutic itineraries of snakebite victims and antivenom access in southern Mexico
Source: PLoS Negl Trop Dis. 2024 Jul 5;18(7):e0012301. doi: 10.1371/journal.pntd.0012301 (PMC11262687; doi:10.1371/journal.pntd.0012301)
Supplement: S1 Interview summaries — (ZIP) [file pntd.0012301.s002.zip › vasquez-neri-carter_2024_data_files/Interview Summaries/Interview Summaries/Ramiro.docx]

Ramiro, [locality name redacted to protect confidentiality], mordido 2019, tenía 46 años

Ramiro tenía 46, estaba cuidando los cafetales en 2019 cuando una cola blanca (*Agkistrodon bilineatus*; *Bothrops asper*) lo mordió en el tobillo. Se hincho su pierna. Camino 30 minutos para llegar a su comunidad en el Ejido [locality name redacted to protect confidentiality], donde tomo café amargo. Fue en moto directamente 30 minutos a Mapastepec en motocicleta y recibió antídoto.

“Tome café amargo nadamas, y llegando al hospital [locality name redacted to protect confidentiality], me inyectaron.”
