# Supplementary material for: Therapeutic itineraries of snakebite victims and antivenom access in southern Mexico
Source: PLoS Negl Trop Dis. 2024 Jul 5;18(7):e0012301. doi: 10.1371/journal.pntd.0012301 (PMC11262687; doi:10.1371/journal.pntd.0012301)
Supplement: S1 Interview summaries — (ZIP) [file pntd.0012301.s002.zip › vasquez-neri-carter_2024_data_files/Interview Summaries/Interview Summaries/Roberto.docx]

Roberto, [locality name redacted to protect confidentiality], mordido 2003, tenía 50 años

Roberto fue mordido en el pie izquierdo por un coralillo mientras cuidaba las plantas de frijol en 2003. Le cortó la herida y le hizo un torniquete. Caminó una hora hasta la clínica de [locality name redacted to protect confidentiality]. De allí lo enviaron a [locality name redacted to protect confidentiality], donde le inyectaron un vial de antídoto. Quedo 3 meses en cama.
