# Supplementary material for: Therapeutic itineraries of snakebite victims and antivenom access in southern Mexico
Source: PLoS Negl Trop Dis. 2024 Jul 5;18(7):e0012301. doi: 10.1371/journal.pntd.0012301 (PMC11262687; doi:10.1371/journal.pntd.0012301)
Supplement: S1 Interview summaries — (ZIP) [file pntd.0012301.s002.zip › vasquez-neri-carter_2024_data_files/Interview Summaries/Interview Summaries/Samuel.docx]

Samuel, [locality name redacted to protect confidentiality], mordido 1997, tenía 9 años

Samuel fue con su tío a trabajar en una plantación de hule cuando tenía 9 años (hace 25 años). Un robahueso (descrito normalmente como de medio metro de largo, de color negro grisáceo) lo mordió en el tobillo y lo sintió casi como una picadura de hormiga. Continuó trabajando, pero unos pasos más tarde, sintió que le pesaba la cabeza. Miró hacia atrás y vio a la serpiente acurrucada en el suelo, lista para morderlo nuevamente. Le mostró a su tío la serpiente y le dijo que sentía la cabeza pesada, y su tío dijo: ¡Te mordió! El tío mató a la serpiente (encontró que el robahueso tenía un coralillo en el estómago) y luego le hizo un torniquete en la pierna. Tomaron el autobús hacia la ciudad, luego se bajaron y empezaron a caminar. Después de media hora, la pierna de Samuel estaba hinchada y no podía caminar. Fueron a ver a Don Pepe, un curandero local en [locality name redacted to protect confidentiality]. Le dio a Samuel una bebida de hierbas amargas que no quiso terminar. El curandero le dijo a Samuel que no lo ayudaría hasta que Samuel terminara la bebida. Don Pepe pinchó el tobillo de Samuel con una aguja para inyectar ganado. El curandero tomó un frasco de mayonesa limpio, le echó un poco de alcohol y le prendió fuego para crear un vacío. Luego, colocó el frasco sobre la herida y salió un líquido amarillo. Según Don Pepe, este método succionaba el veneno. Don Pepe echó agua caliente sobre el tobillo luego del proceso de succión, al que llaman “ventosa”. Don Pepe repitió este proceso todos los días durante aproximadamente una semana y media. También oraba/cantaba todos los días y Samuel se rió una vez. Don Pepe reprendió a Samuel por reírse. Dejó de ir a la escuela después de la mordedura de serpiente.

Samuel sabía de Don Pepe porque su padre era del mismo pueblo.

“Aquí hay varios que les pico la culebra. Yo estaba chapeando hule, y sentí que me pica, casi duele más una hormiga. Cuando sentí ese piquetito, seguí chapeando, pero a los dos pasos más, sentí que la cabeza me pesaba. Y voltee atrás, y ahí estaba enrollada. Ya estaba para darme otra. Eso le dije a mi tío, ‘¡Una culebra me pico!’ Y me dice, ‘Si, ¡se te pico!’ Me dijo ‘vete corriendo. Corre y dile a tu papá que te pico la culebra.’ Y él se quedó para matarlo. Y llego y lo que hace ellos, me amarraron con una liga. Me amarraron el pie. Y me llevaron. En ese tiempo había autobús, carro, y en ese momento podía caminar. Pero bajando del carro a la media hora, ya no camine. Ya me cargaron al que curaba piquete de culebra, a su casa. Antes de meter la mano, me dio la bebida. Amarguisima! Yo lloraba. Me dijo ‘Aquí hasta que acabes este vaso, voy a meter mano para curarte.’ Era una toma. Lo tomé, lo tomé y lloré. Hasta que lo acabe. Lo que hizo este don, me puso el pie arriba. Puso ventosa. Es un trastito de mayonesa, lo echas alcohol, y lo enjuaga y lo seca, y hecho una hoja en la herida mientras que echó alcohol. Una vez que hecho el alcohol, agarro un cerillo, prendio y vvvt! Con un dedo jalo la hoja y bah! Y ahí agarró. [ruido de succión] Eso saco el veneno, la ventosa. Cuando ya salía sangre pura, es porque ya el veneno había salido. Y temblaba mi pie porque estaba la hoja ahí. Y me hizo casi una semana y media, dos semanas que me estaba curando con esa ventosa. Después de esa curación, ya me puso agua caliente. Y era de diario que lo curaba. Y acabando la curación, hizo como todos los culebreros, hacía su oración. El declaraba sano. Y yo me reí, y me regaño. Después me fui a la casa. Me cobró 500 pesos.”

“El coralillo casi no pica a la gente. Y cuando pica, es mortal. Esa culebra, el robahueso, comió al coralillo. Porque lo mató mi tío y lo tenía en su panza. El robahueso es igualito como una sorda pero es chica, siempre es medio metro no más, máximo como un metro. No puede crecer más. Es color gris.”

“Cuando me pico la culebra ya no fui a la escuela. Cuando el clima cambia, me duele el pie. Cuando viene Noviembre me duele.”

“En ese entonces no había clínica, nada para la culebra. Era valor mexicano, si tu sabias que hay curandero de culebra, lo curaba. Ellos hacen un pacto. Cada Semana Santa, llaman a la culebra. Desde el más chiquito hasta el más grande. Ya no son gente normal, pero curaban bien. Si no viene ese, la gente se moría.”
