# Supplementary material for: Therapeutic itineraries of snakebite victims and antivenom access in southern Mexico
Source: PLoS Negl Trop Dis. 2024 Jul 5;18(7):e0012301. doi: 10.1371/journal.pntd.0012301 (PMC11262687; doi:10.1371/journal.pntd.0012301)
Supplement: S1 Interview summaries — (ZIP) [file pntd.0012301.s002.zip › vasquez-neri-carter_2024_data_files/Interview Summaries/Interview Summaries/Santiago.docx]

Santiago, [locality name redacted to protect confidentiality], mordido 1997 y 2005, tenia 11 y 19 años

1. Santiago, hombre Tseltal de [locality name redacted to protect confidentiality], tenía 11 años en 1997, cuando él y su hermano caminaban entre los árboles alrededor de su casa para traer leña para el fuego familiar. De repente, Santiago fue mordido en la pantorrilla por una serpiente. Dice Santiago que fue una “chichicua”, *Spilotes pullatus*, una especie no venenosa. Unos minutos más tarde, se sintió mareado y con náuseas. Su pierna se hinchó. La hermana de Santiago lo llevó al centro del pueblo para consultar al curandero local. El curandero rompió una botella vieja, abrió la herida y succionó el veneno. Le dio a beber a Santiago unas semillas en alcohol. Con el tiempo, la inflamación disminuyó, y pudo caminar 3 días después de su accidente. No fueron al hospital porque el hospital más cercano estaba lejos.
   1. “Lo vamos a curar con este señor grande [el curandero], porque de aquí hasta que llega a [locality name redacted to protect confidentiality] me iba a morir.”
   2. “Agarramos una botella… lo rompió [el curandero] y me corto bastante. Lo empezó a chupar y chupar y chupar para que salga el veneno.”
   3. “Segun lo que me dicen, no fue venenosa esa culebra que me pico. Si fuera muy venenosa tal vez hubiera muerto porque de aquí al hospital, ya… está retirado.”
   4. “esta bueno acudir al hospital porque dan buenos medicamentos.
2. Ocho años después, Santiago tenía 20 años y vivía en [locality name redacted to protect confidentiality]. Santiago estaba trabajando, moviendo piedras y construyendo un camino cerca de su casa en [locality name redacted to protect confidentiality] cuando sintió la mordedura en la parte posterior de su pie. No logró identificar a la serpiente, como era de noche, pero vio la cola y dice que sintió la mordedura “como piquete de avispa o aguja, como una espina”. Se desmayó 10 minutos después y se despertó en el hospital a las 11 de la mañana. Su hermano lo llevó unos 15 minutos al hospital. Estuvo en el hospital durante aproximadamente 3 días, le salió sangre de la nariz, los ojos y los oídos durante 2 semanas. Su pierna todavía está débil.

En general, Santiago se siente más cómodo en el hospital porque tienen más herramientas para curar las mordeduras de serpientes venenosas. ”El hospital está lejos pero tiene buena medicina.”
