# Supplementary material for: Therapeutic itineraries of snakebite victims and antivenom access in southern Mexico
Source: PLoS Negl Trop Dis. 2024 Jul 5;18(7):e0012301. doi: 10.1371/journal.pntd.0012301 (PMC11262687; doi:10.1371/journal.pntd.0012301)
Supplement: S1 Interview summaries — (ZIP) [file pntd.0012301.s002.zip › vasquez-neri-carter_2024_data_files/Interview Summaries/Interview Summaries/Silvestre.docx]

Silvestre, [locality name redacted to protect confidentiality], mordido 2021, tenía 60 años

Silvestre fue mordido en 2021 por robahueso. Estaba bajando de su barco pesquero, a la orilla del río. En su primer paso fuera del barco pisó el robahueso, y la serpiente le mordió en el dedo medio del pie. Sintió una picadura como la de una hormiga y mató a la serpiente. Le dijo a un transeúnte que lo habían mordido. El transeúnte ató un torniquete alrededor de la pierna de Silvestre y le dio de comer limonada y ajo crudo. El sobrino de Silvestre llevó a Silvestre al hospital [locality name redacted to protect confidentiality]. Llegaron al hospital unos 30 minutos después, pero el hospital no tenía el antídoto. La familia de Silvestre fue a buscar antídoto y le compraron dos a un médico privado por 3500 pesos cada uno. Querían comprar más, pero el doctor sólo tenía dos. A Silvestre le inyectaron los antivenenos aproximadamente una hora y media después de haber sido mordido. Estuvo en el hospital durante 3 días. Silvestre habla chinanteco.

“Andaba yo pescando, y después de la pesca salí. Y estaba una serpiente, y di el paso y ahí estaba el animal, mero donde cayó el pie. Me mordió el dedo del pie. Sentí un piquete como una hormiga. Quedó entumida, como electricidad. Y lo vi y le dije 'Tú fuiste lo que me chingaste’ y lo maté. Enseguida un vecino me amarró el pie, bien amarrado. Me dio un vaso de agua de limón, me dio un ajo para masticar. Corrió la gente para ayudarme porque esa culebra es muy venenosa. Un coche de mi sobrino me llevó al hospital. Pero ahí no tenían la medicina adecuada para matar el veneno. No lo tenían entonces mi familia lo consiguió. Me pusieron 2, y dijeron que me iban a poner 3 pero nada más consiguieron 2. Ya no hubo más. Me lo dieron en 3,500 pesos. Es caro. Deben de tenerlo en el hospital en casos así.”

“En la siembra de café hay muchos casos. Les muerde varios. Y si le muerde la cabeza ya no hay remedio.”
