# Supplementary material for: Therapeutic itineraries of snakebite victims and antivenom access in southern Mexico
Source: PLoS Negl Trop Dis. 2024 Jul 5;18(7):e0012301. doi: 10.1371/journal.pntd.0012301 (PMC11262687; doi:10.1371/journal.pntd.0012301)
Supplement: S1 Interview summaries — (ZIP) [file pntd.0012301.s002.zip › vasquez-neri-carter_2024_data_files/Interview Summaries/Interview Summaries/Vicente.docx]

Vicente, [locality name redacted to protect confidentiality], mordido 1985 y 1990, tenía 22 y 27 años

Vicente fue mordido dos veces por serpientes venenosas. El primer accidente ocurrió cuando tenía 22 años en 1985, estaba limpiando el cafetal cuando fue picado en la mano por una cola blanca (*Agkistrodon bilineatus* o *Bothrops asper*). Fue serio. Bebió aguardiente. Había un médico tradicional llamado Santiago que vivía al lado. Cuando las serpientes mordían a la gente, preparaba medicinas con hierbas como la curarina y el cedrón, que traía de las montañas.

“Se dio grave pues, y le siguieron picando culebras cada 15 días. Siempre estaba trabajando en el potrero, limpiando café. A mi hermano pasaban casos y casos y casos. Pero tomó aguardiente. El trago es bueno para eso, el trago es caliente. Se emborrachaba bien. Tomo café caliente, amargo. Pero vive un hombre aquí que tenía remedio preparado para la serpiente. Este cedrón, curarina,... hay muchos de estos en el monte.”

“La picadura de cantil deja 4 marcas de colmillos.”

Posteriormente Vicente fue mordido por tamagaz, y cantil. Fue mordido un total de 12 o 13 veces, siempre cuando trabajaba en plantaciones de café o maíz. Bebía alcohol cada vez porque el alcohol es picante, con cedrón y curarina. Siempre se sintió bien después. Hizo dietas especiales, evitando aceites y grasas tras la picadura.

“Tan repente la desgracia. No sabemos, la vida es así.”

“[El curandero] nos decía que no comía caldo de pollo, que no comía frijoles con huevos.”
