# Supplementary material for: Therapeutic itineraries of snakebite victims and antivenom access in southern Mexico
Source: PLoS Negl Trop Dis. 2024 Jul 5;18(7):e0012301. doi: 10.1371/journal.pntd.0012301 (PMC11262687; doi:10.1371/journal.pntd.0012301)
Supplement: S1 Interview summaries — (ZIP) [file pntd.0012301.s002.zip › vasquez-neri-carter_2024_data_files/Interview Summaries/Interview Summaries/Victor.docx]

Victor, [locality name redacted to protect confidentiality], mordido 2019, tenía 31 años

Víctor tenía 31 años y trabajaba en la finca cafetalera en Febrero de 2019 cuando fue mordido por una “cotorrera” o *Bothriechis bicolor*, en la mano derecha. Caminó una hora hasta su casa y luego al centro de salud Compañeros de Salud. En Compañeros de Salud, le dieron a Víctor solución salina y lo trasladaron al hospital de [locality name redacted to protect confidentiality], un viaje de cuatro horas. En [locality name redacted to protect confidentiality] le inyectaron antiveneno (aunque no recuerda cuántas ampollas). Permaneció internado en el hospital de [locality name redacted to protect confidentiality] durante 3 días. Ya no tiene dolor.

“Cuando me iba a meter ahí al río, al meterme al agua, el color de mi piel empezó a cambiar al color de la culebra por el frío del agua, o no se.”
