# Supplementary material for: Therapeutic itineraries of snakebite victims and antivenom access in southern Mexico
Source: PLoS Negl Trop Dis. 2024 Jul 5;18(7):e0012301. doi: 10.1371/journal.pntd.0012301 (PMC11262687; doi:10.1371/journal.pntd.0012301)
Supplement: S1 Interview summaries — (ZIP) [file pntd.0012301.s002.zip › vasquez-neri-carter_2024_data_files/Note.docx]

**Article information:**

Vasquez, C, E Neri Castro, & ED Carter (2024). Therapeutic itineraries of snakebite victims and antivenom access in southern Mexico. *PLOS – Neglected Tropical Diseases*. Conditional acceptance received June 19, 2024.

**File contents:**

1. **Interview Summaries – Excel**. Excel spreadsheet file, with information on snakebite victims recorded for the project. Pseudonyms are used for informants. Locality names have been redacted to protect confidentiality of informants.
2. **Interview Summaries (folder)**. Contains 47 Word documents, each summarizing an interview for the project. Mainly in Spanish. Pseudonyms are used for informants. Locality names have been redacted to protect confidentiality of informants.
