# Supplementary material for: Therapeutic itineraries of snakebite victims and antivenom access in southern Mexico
Source: PLoS Negl Trop Dis. 2024 Jul 5;18(7):e0012301. doi: 10.1371/journal.pntd.0012301 (PMC11262687; doi:10.1371/journal.pntd.0012301)
Supplement: Attachment — Submitted filename: cover_letter_revised_after_technical_check.docx [file pntd.0012301.s003.docx]

15 May 2024

Dear Editors of PLOS-Neglected Tropical Diseases:

Please find enclosed the revised version of the manuscript entitled "Therapeutic itineraries of snakebite victims and antivenom access in southern Mexico," which we are resubmitting to be considered as a research article for the journal *PLOS-Neglected Tropical Diseases*.

We found the reviewers' comments to be very helpful, and we have revised the manuscript according to their suggestions. Here is a list of our responses to reviewers (we denoted our responses with *** and bold type). We only responded to comments that required some action from us.

PLOS-NTDs

Reviewer's Responses to Questions

Key Review Criteria Required for Acceptance?

As you describe the new analyses required for acceptance, please consider the following:

Methods

-Are the objectives of the study clearly articulated with a clear testable hypothesis stated?

-Is the study design appropriate to address the stated objectives?

-Is the population clearly described and appropriate for the hypothesis being tested?

-Is the sample size sufficient to ensure adequate power to address the hypothesis being tested?

-Were correct statistical analysis used to support conclusions?

-Are there concerns about ethical or regulatory requirements being met?

Reviewer #1: This study is descriptive and does not aim to prove or disprove a specific hypothesis. In light off this, I believe it is of qualitative interest to understand the hurdles that snakebite victims face in the study region.

Reviewer #2: This is a funny but well-articulated manuscript that discusses many important considerations in snake envenomation not only in rural Mexico but also worldwide, particularly in rural South America, Africa, and Asia.

Reviewer #3: The hot and cold system therapeutic framework has been described to explain practices around snakebites. In this way, some additional data of those living in Sierra Madre and Chinantla Baja regions would be useful to understand their cultural or anthropological background. Was there any difference in the perceptions of snakebite risk, use of traditional medicine or confidence in antivenom treatment?

***** Thank you for this comment. We understand what the reviewer is asking of us, but it's difficult to get into additional anthropological analysis of the cultures of the Sierra Madre and Chinantla Baja regions, due in part to our lack of data. I wish we could draw stronger connections between elements of local culture, the hot-cold theory, and the therapeutic itineraries of snakebite victims, but we are not able to do so, in depth, in this article. However, the last paragraph of the Discussion (lines 533-550) have been revised in such a way that some of the reviewers' concerns here might be allayed.**

Interviewing victims of snakebites in their lifetime might cause recall bias. Authors noted the risk of cognitive bias associated with mistaken self-diagnosis of snakebite. However, no there were comments regarding the possibility of distorted or inaccurate memory of their therapeutic itineraries. In which extension, their responses might not accurately represent the reality? Has any strategy been implemented to lessen its effects on data accuracy to mitigate recall bias in the reports?

***** Thank you for this important comment. We've updated the manuscript in two ways to respond to this critique. First, in the Results section, we have added about 1 paragraph of new analysis (with associated tables and figures), that discusses the differences in treatment-seeking between groups that experienced snakebite in early (1977-2010) versus later (2011-2023) periods. Disaggregating the data by time of snakebite could help to address the reviewer's concern (lines 373-381). Second, we also added a note about recall bias in a paragraph on study limitations, in the paper's Conclusion (lines 569-571).**

--------------------

Results

-Does the analysis presented match the analysis plan?

-Are the results clearly and completely presented?

-Are the figures (Tables, Images) of sufficient quality for clarity?

Reviewer #1: Yes, I only suggest presenting some of the results as figures, instead of in-line numerical descriptions.

***** Thank you for your recommendation! Figure 2 and figure 3 (around lines 197-202) present this information in graphic form.**

Reviewer #2: Yes, they are.

Reviewer #3: As observed in other studies, the storytelling methods provided a very illustrative scenario of the difficulties to the accessibility of antivenom. Tables and photos are sufficiently clear and contributed for a better understanding of the results.

Distance was referred as a barrier to antivenom access, but the mean time to reach a hospital was less of three hours (although considerable standard deviation). If better roads and improvements in the public health system might be associated with reduction of the distance, in which proportion other variables contributed to the perception of barriers in accessing antivenom? It is not clear if the use of traditional medicine at the community would be considered a cause or consequence of the limitation of transportation for the time to reach hospital.

***** Thank you for this comment. It seems like traditional medicine was both a cause and consequence of long travel times. We have added additional discussion and clarification in the Discussion, around lines 523-530.**

There is no information regarding the health policy in the country and how antivenom is distributed. Should antivenom treatment be always paid by the patient? Does it mean the public health system in Mexico does not cover antivenom treatment? Please, convert price of antivenom (3,500 pesos per vial) from Mexican currency to American dollar.

***** Prices have been converted to dollars. We have added a paragraph about Mexican health care policy, including the structure of the Mexican health care system, and how it affects quality of care and antivenom access more specifically, around lines 511-522 in the Discussion.**

--------------------

Conclusions

-Are the conclusions supported by the data presented?

-Are the limitations of analysis clearly described?

-Do the authors discuss how these data can be helpful to advance our understanding of the topic under study?

-Is public health relevance addressed?

Reviewer #1: I am not a social scientist, so as far as my understanding of the study I don't have any major concerns.

Reviewer #2: Yes, they are.

Reviewer #3: Some more discussion is lacking about the confidence in the antivenom treatment, which was noticeable, and with no conflict with traditional medicine.

***** We have tried to respond to this concern with an expanded discussion of why biomedical/allopathic and traditional medicine are integrated in practice, in the Discussion, lines 533-550.**

Also, could the hot and cold theory contribute to the recognition of the relevance and intercultural applicability of these medicines, both in the clinical, academic, and political settings?

***** In the same part of the Discussion, around lines 543-545, we explain how the hot-cold theory exemplifies a cultural aspect of health-seeking behavior that should be considered within an intercultural approach, and here we also advocate for the usefulness of the intercultural approach.**

--------------------

Editorial and Data Presentation Modifications?<br/>

Use  this section for editorial suggestions as well as relatively minor modifications of existing data that would enhance clarity. If the only modifications needed are minor and/or editorial, you may wish to recommend “Minor Revision” or “Accept”.

Reviewer #1: Comments on: Therapeutic itineraries of snakebite victims

I found the acrticle very well written and a very valuable descriptive study. I feel however that the findings, ranging from qualititive to purely quantitative would benefit from putting into a wider context (see Longbottttom et al. 2018. The Lancet. <https://doi.org/10.1016/S0140-6736(18)31224-8>).

***** In the revised version, we refer to this study (Longbottom, et al.) in the Conclusion, around line 560. Although we aren't able to offer a full critique of this study, we contend that it may underestimate the vulnerability of rural people in places like southern Mexico, to the extent that it does not consider the complexities of actually finding and obtaining antivenom treatment.**

The results section, Characteristics of snakebite victims and Post-bite practices, would benefit from including some graphics, especially those which highlight the study's aims such as frequency of treatments sought. Pie charts would help communicate these findings.

***** Thank you for the suggestion. Figure 2 and figure 3 (around lines 225-232) present this information in graphic form.**

Reviewer #2: Acknowledgments:

Line 561. "I also thank", who is I?

Line 566. "who accompanied me", who is me?

***** Acknowledgement section has been updated and simplified.**

Reviewer #3: Line 23 – Add neuroparalytic effects when describing the variety of symptoms and associated deaths, as elapids maybe also a cause of morbidity and mortality.

Line 112 – Metlapilcoatlus occiduus should be typed in italic.

***** Metlapilcoatlus occiduus italicized on line 148, neuroparalytic effects added to the description of symptoms and associated deaths on line 63.**

--------------------

Summary and General Comments

Use  this section to provide overall comments, discuss strengths/weaknesses of the study, novelty, significance, general execution and scholarship. You may also include additional comments for the author, including concerns about dual publication, research ethics, or publication ethics. If requesting major revision, please articulate the new experiments that are needed.

Reviewer #1: The main weakness is the small sample size. They make up for this with a lot of information from each interviewee, but some comparison of experiences between study sites may be of value to understand how the small sample size may affect their conclusions.

***** Around lines 434-444 of the Results section, we make some comparison between the Oaxaca and Chiapas study sites, particularly how in Oaxaca Chinantla Baja a local culebrero played a role in administering treatments, unlike Chiapas. But, as the reviewer is implying, the sample size for Oaxaca is too small for us to make other, more systematic comparisons between experiences there and in Chiapas.**

Reviewer #2: I think is an interesting manuscript and has value to be published.

Reviewer #3: There is a tendency to use qualitative methods to collect data and discuss the results in studies referring snakebite envenoming in traditional groups, as this interesting study of the therapeutic itineraries of patients bitten by snakes to reach antivenom treatment in Southern Mexico.

In response to a technical check (email from PLOS dated May 10), we made several other changes, including addition of supplementary files, removal of one image, and addition of author summary after abstract.

As before, the manuscript is not under review elsewhere. It will not be submitted to any other publication while it is under consideration by *PLOS-Neglected Tropical Diseases*. The contents have not been published elsewhere in any form, and the text is not under copyright. We are the sole authors of the manuscript.

We hope that the editors and the reviewers will agree that we have responded to the reviewers' concerns, and that the manuscript is now suitable for publication.

Sincerely (on behalf of co-authors),

Eric D. Carter

Edens Professor of Geography and Global Health

Macalester College (Minnesota, USA)
